# Naturally occurring prenylated flavonoids from African *Erythrina* plant species†

**DOI:** 10.1039/d5ra03457d

**Published:** 2025-08-18

**Authors:** Bienvenu Tsakem, Fidele Ntie Kang, Rémy Bertrand Teponno, Xavier Siwe Noundou

**Affiliations:** a Department of Pharmaceutical Sciences, School of Pharmacy, Sefako Makgatho Health Sciences University Pretoria 0204 South Africa xavier.siwenoundou@smu.ac.za; b Center for Drug Discovery, Faculty of Science, University of Buea P.O. Box 63 Buea Cameroon; c Department of Chemistry Faculty of Science, University of Buea P. O. Box 63 Buea Cameroon; d Institute of Pharmacy, Martin-Luther University Halle-Wittenberg Kurt-Mothes-Strasse 3 06120 Halle Saale Germany; e Department of Chemistry, Faculty of Science, University of Dschang P.O. Box 67 Dschang Cameroon

## Abstract

Flavonoids refer to a large class of secondary metabolites with a unique skeleton comprising two aromatic rings linked together by a C_3_ unit. This class of compounds is largely distributed in plants and rarely occurs in fungi. Some of these compounds are directly bonded to prenyl units. Prenyl groups are C-5 carbon units derived from the mevalonate pathway, are reported to substantially improve the biological activities of diverse classes of secondary metabolites. Prenylated flavonoids display a broad spectrum of biological activities, including antibacterial, antioxidant, anticancer, antidiabetic and antiviral activities. Some flavonoids have already been formulated as either medicines or dietary supplements and are currently used in the management of certain medical conditions. The continuous search for bioactive molecules is a global concern; and encapsulating the contribution of each continent and/or country in terms of available resources should be a priority. This paper aims to methodically summarize the bioactive prenylated flavonoids characterized from plants of the genus *Erythrina* growing in Africa, as well as their distribution in the genus. Approximately 289 prenylated flavonoids have been isolated and characterized exclusively from plants belonging to the genus *Erythrina* growing in Africa, covering all the subclasses of flavonoids bearing prenyl group(s), namely, flavanones, flavones, chalcones, isoflavanones, isoflavones, isoflavans, isoflav-3-enes, pterocarpans and pterocarpenes isolated from 1981 to date. This review encompasses the data gathered from 202 peer-reviewed articles and covers the source, isolation, distribution of *Erythrina* plant species throughout the continent, structure elucidation of prenyl moieties, biological activities as well as the *in silico* tests where available towards some targets in drug discovery.

## Abstract 2

This paper provides a systematic overview of the bioactive prenylated flavonoids isolated from African species of the genus Erythrina, along with their distribution within the genus.

## Graphical abstract

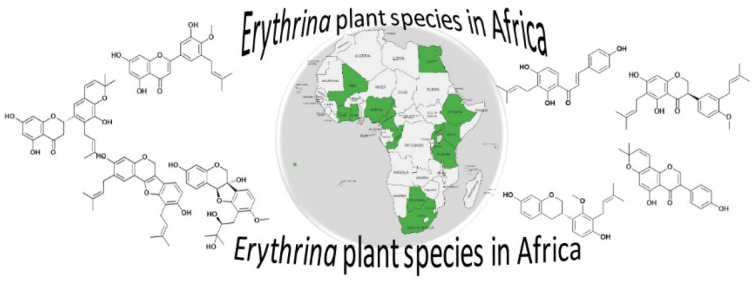

## 1 Introduction

The search for new hits and leads from any source of secondary metabolites, including plants, fungi, and bacteria, has been a focus of interest for chemists and pharmacists over the last few decades.^1–5^ There is a continuous need to discover novel and more effective active principles, particularly during the emergence of new diseases and infections. This includes the severe acute respiratory syndrome coronavirus 2 (SARS-CoV-2), the drug resistant phenomena, as well as the existence of diseases without known or effective treatments, such as HIV-AIDS and many forms of cancer.

Natural products such as flavonoids are of paramount importance in drug discovery. Prenylated flavonoids are synthesized by plants as a response to microbial attacks.^6^ Interestingly, these natural compounds are reported to exhibit improved biological activities compared to the original skeleton without the prenyl groups.^7^ The increase in biological activities of prenylated flavonoids is generally ascribed to the increase of their lipophilicity due to the presence of prenyl moieties.^7^ The penetration of prenylated flavonoids into cells is facilitated due to the fact that the cell membrane of microbes is phospholipidic.

While several African species of the *Erythrina* genus have been extensively investigated, and have resulted in reports of a number of prenylated flavonoids,^8,9^ there is no available report documenting the significance of prenylated flavonoids in these African plant species from the *Erythrina* genus, or their distribution within the genus in Africa. There have been two review articles world-wide on non-alkaloidic compounds of the genus *Erythrina*, published in 2005 (ref. 10) and 2018.^11^ Further, there is one review article, published in 2021, summarizing all isolated flavonoids in general from any investigated *Erythrina* species,^12^ while a recent review in 2023 (ref. 13) reports on prenylated flavonoids from numerous plants, however, this only mentioned 10 species of *Erythrina*, while about forty species throughout the world have been already studied for their content in prenylated flavonoids.

**Fig. 1:**
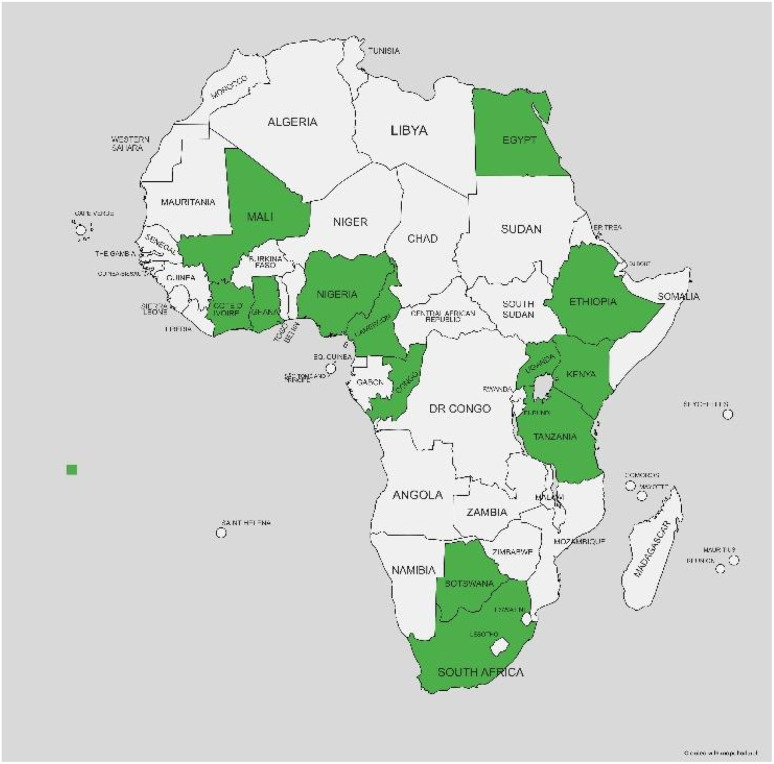
Distribution of studied *Erythrina* species in Africa.

Considering all the benefits of flavonoids to human health for cancer management, inflammation and immune system boosting, some of these molecules could be beneficial for further medical conditions such as COVID-19, AIDS, breast cancer, to mention a few. Unfortunately, these natural products are usually isolated in small amounts and sometimes biological assays are not conducted. Some flavonoids have just been submitted to *in vitro* assays, while others have been reported just from one source. Given that all these data are completely dispersed, this review is focussed only on prenylated flavonoids previously isolated from all *Erythrina* species distributed in Africa. The focus on African *Erythrina* plants has three objectives. Firstly, any information on the prenylated flavonoids in African *Erythrina* will be highlighted; secondly, as all information will be contained in a single document, it will facilitate the search for information regarding *Erythrina* genus in Africa, as shown in Fig. 1, and finally the review highlights the contribution of African plants as a source of potential lead compounds in drug discovery. It is worth mentioning that non-prenylated and prenylated flavonoids are currently used as medicines and dietary supplements.^14–16^

The aim of this review is to methodically summarize the bioactive prenylated flavonoids isolated during the period 1981 to 2024 from plants of the genus *Erythrina* growing in Africa, by providing their source, isolation, distribution, structure elucidation and biological activities, as well as their drug target analysis.

## 2 Prenylated flavonoids

### 2.1 Definition

**Fig. 2:**
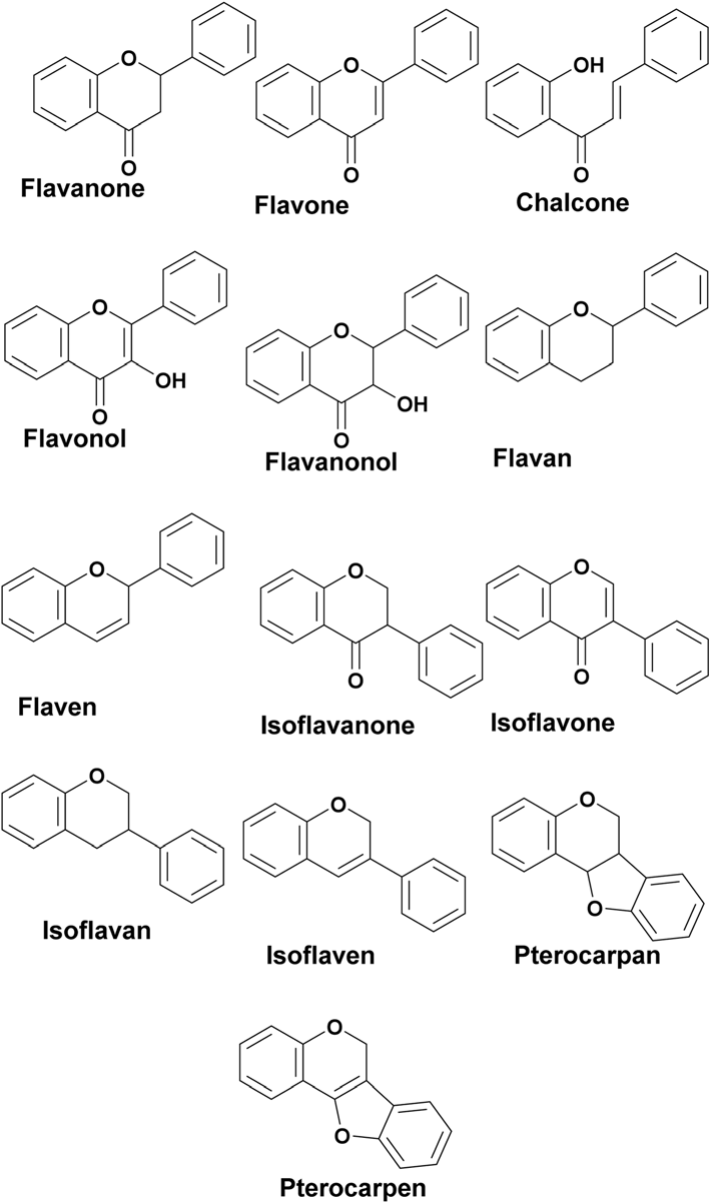
Common skeletons/subclasses of flavonoids.

Flavonoids are a group of plant polyphenolic secondary metabolites featuring a common three-ring chemical structure C_6_–C_3_–C_6_ core (diphenylpropane derivatives). They consist of a skeleton containing two benzene rings connected by a C_3_ moiety. The first C_6_ ring is called the A ring, the second is called the B ring, and the C_3_ moiety can either be opened (forming an aliphatic chain) or closed (forming a six-membered ring attached to A ring) and is called the C ring.^17^ Flavonoids with a C-ring are also called benzopyran.^18^ Relative to the position of the linkage of the B ring to the benzopyran (chromano) moiety, flavonoids may be divided into thirteen sub-classes grouped as 2-phenylbenzopyrans (flavanones, flavones, chalcones, flavonols, flavononols, flavanes, flavens), isoflavonoids or 3-phenylbenzopyrans (isoflavanones, isoflavones, isoflavanes, isoflavens, pterocarpans, pterocarpens) and neoflavonoids or (4-phenylbenzopyrans) (Fig. 2).^18^

### 2.2 Biosynthesis

Taking into consideration that the biosynthesis of flavonoids in general has been reported,^19,20^ we focus here on the prenylation, which, while it would seem to be a causal mechanism, there are a lot of specificities within flavonoid subclasses. Depending on the genes encoding for the biosynthesis of flavonoids in plants, some may carry a prenyl on the rings. Shi *et al.* in 2021 designated prenylated flavonoids as flavonoids that associate one or more prenyl groups linked either by C–C bond(s) or C–O bond(s) to their rings.^7^ The so-called prenyl groups derive from the dimethylallylpyrophosphate (DMAPP), resulting from the condensation of 3 units of acetyl coenzyme A.^21^

According to the report of Yazaki and coworkers, membrane-bound prenyltransferases which accept aromatic substrates are sub-divided into two main groups, namely, *p*-hydroxybenzoate (PHB) prenyltransferases and homogentisate (HG) prenyltransferases. Flavonoid prenyltransferases are derived from the HG prenyltransferase family.^22^ These enzymes are located in the plastid in plant cells^23^ and their catalytic action in the presence of a cofactor, Mg^2+^ is reported to be the best cofactor in these reactions.^22^

In an enzyme assay, dimethylallyl diphosphate (DMAPP) and the enzyme SfN8DT-1 identified in *Sophora flavescens* (naringenin 8-dimethylallyltransferase) were found to be specific to flavanone as a substrate.^24^ A paralogue flavonoid prenyltransferase SfFPT from *S. flavescens* (93% SfN8DT-1) has been shown to be non-specific only to flavanones, it also catalyzes the prenylation of flavones, flavanonols and even chalcones. It is worth mentioning that the amount of prenylated flavonoid is lower than that of flavanone as a substrate,^6,25^ and it only inserts a prenyl group at C-8, while when the hydroxyl at 7 is methylated or glycosylated, there is no prenylation.^6^ This might suggest the participation of this hydroxyl in the biosynthesis. The recombinant indole prenyltransferase 7-DMATS, identified from the fungus *Aspergillus fumigatus*, catalyzes C-6 prenylation in chalcones, isoflavonoids and flavanones. The enzyme AnaPT, equally identified in *Aspergillus fumigatus* has shown its ability to prenylate to the C-3′ position of chalcones.^26–28^

**Fig. 3:**
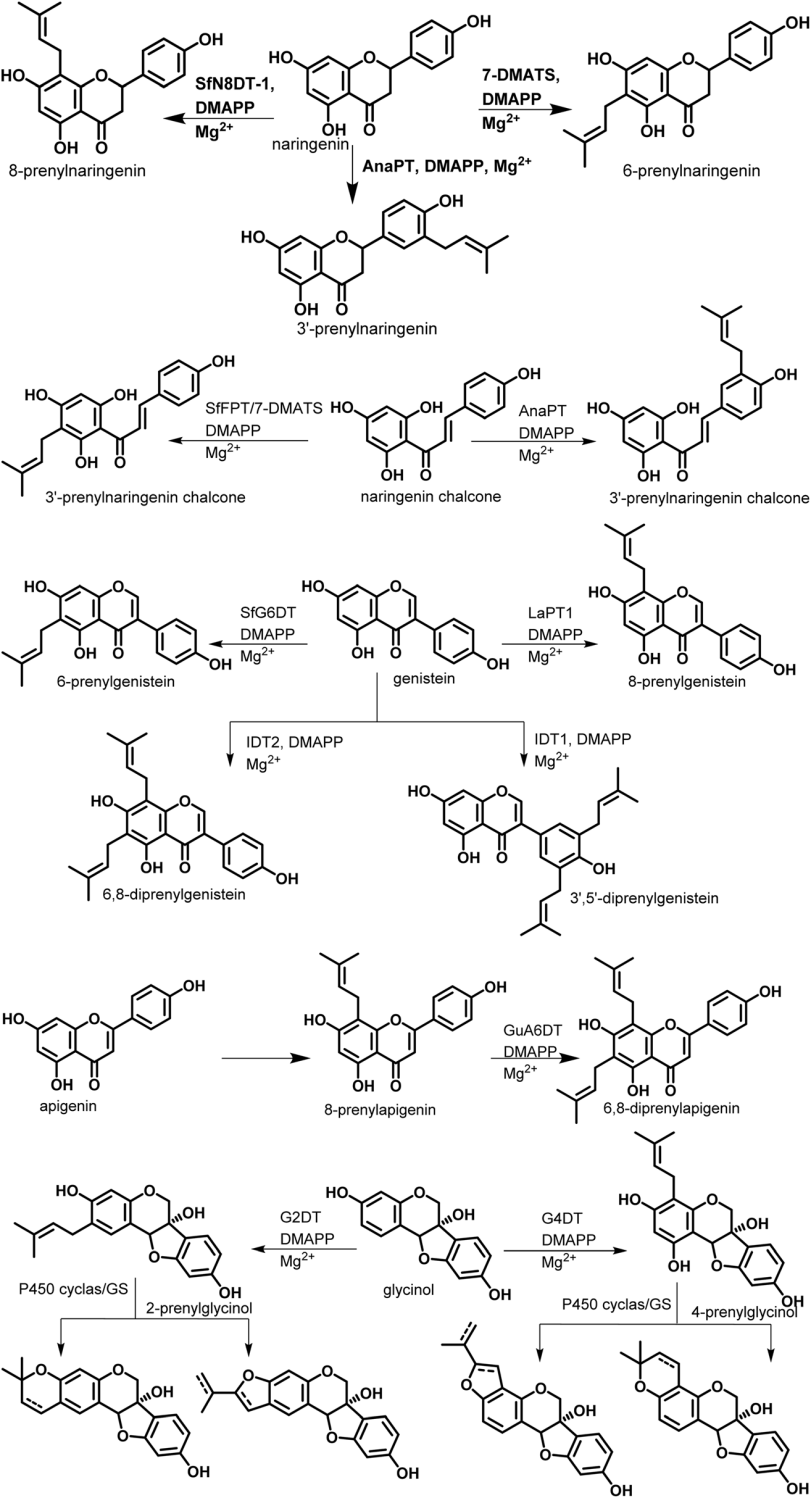
Biosynthetic routes of prenylation of flavonoids.

Pterocarpan subclass possesses a specific prenyltransferase which shares 50% of significant similarity with SfN8DTs. The gene has been identified previously in soybeans and was given the name GmG4DT,^29–31^ or more specifically GmPT20.^32^ It attaches DMAPP at C-8 of the native pterocarpan substrate, glycinol and the reaction is catalyzed by G4DT, while GmPT20 encodes for the prenylation on C-6 catalyzed by G2DT. Further cyclization reactions in these prenylated pterocarpan are catalyzed by glyceollin synthase (GS) or P450 cyclase.^30^ The enzyme SfG6DT inserts a prenyl group in genistein on carbon 6 and LaPT1 prenylate the B-rings in isoflavone such as genistein and 2′-hydroxygenistein. The chalcone-specific prenyltransferase SfiLDT has been shown to prenylate isoliquiritigenin.^6^ The prenyltransferase GuA6DT identified from *Glycyrrhiza uralensis* specifically introduces a prenyl group at position 6 of flavones; further studies by these authors showed no prenylated derivative in flavonol and flavone, with no hydroxyl groups at C-5 and C-7 or methoxyl group at C-7. Fig. 3 shows the biosynthetic routes of prenylations.

### 2.3 Importance of flavonoids in drug discovery

Several products containing natural molecules, usually referred to as dietary supplements, are progressively integrated in drug markets. Numerous medical conditions are of concern currently despite ongoing efforts in drug discovery and development. Synthetic molecules,^33,34^ natural products from plants and microoganisms^1,5,35,36^ have been at the centre of interest. Ebselen is an approved synthetic drug used as an antioxidant and anti-inflammatory and as a cytoprotective agent, however the same molecule has demonstrated toxicity towards normal cells.^37^ This is just one example amongst others, and this is the reason why some researchers in drug discovery focus on natural products. Numerous molecules from plants are currently used for their therapeutic properties worldwide, which include but are not limited to alkaloids, flavonoids, amino acid derivatives and terpenoids.^38–41^

Some reviews have already reported the implication of flavonoids in cancer evolution and their effects on the nervous system, as well as their antioxidant and anti-inflammatory effects.^38,42–44^ For example, three prenylated flavonoids *i.e.* glabridin, tephropurpurin, and 8-prenylnaringenin have been reported for their benefits in the management and prevention of cancers. Glabridin inhibits the activity of the enzyme CYP3A4 (Cytochrome P450 3A4), the largest class of CYP enzymes. This enzyme is expressed in the human liver and gastrointestinal tract and is involved in the metabolism of 50% of therapeutic agents, as well as in the activation of toxic and carcinogenic substances.^42^ This molecule is sold as a dietary supplement to lighten the skin, and is equally marketed as an anti-inflammatory, antibacterial and pro-apoptotic drug worldwide.^45^ Tephropurpurin induces the activity of NAD(P)H:quinone oxidoreductase, which results in the detoxification of carcinogens. The flavonoid 8-prenylnaringenin inhibits the enzyme CYP1A2 (Cytochrome P450 1A2) which mainly metabolizes important drugs such as phenacetin, theophylline, caffeine, imipramine and propranolol, and also converts some procarcinogens into carcinogens.^42^ Dietary supplements labelled as citrus bioflavonoids made of rutin and ascorbic acid, are consumed to boost the immune system; Nutrivein tart cherry® is marketed as a dietary supplement to relieve pains and for muscle recovery.^45^ Other dietary supplements include tart cherry®, Lipo-flavonoid plus®, Super flavonoids herbal supplement, Ester-C and flavonoids®, citrus bioflavonoids complex® and Bio-flavonoids®.^45^

Many other flavonoids such as quercetin, rutin, naringerin, equol and baicalein have been documented for their inhibition and induction effects on different enzymes contributing to cancer development. Lemos *et al.* reported in 2006 the vasorelaxant activity of floranol, a prenylated flavonoid from the roots of *Dioclea grandiflora*.^46^ Xanthohumol is a prenylated chalcone from *Humulus lupulus* L.; it is the active principle of the dietary supplement equally named Xanthohumol®. This molecule helps to fight oxidative stress and maintain cells in optimal health, promotes drowsiness and calms nervous tension and agitation, as well as supporting hormone balance.^47^

**Fig. 4:**
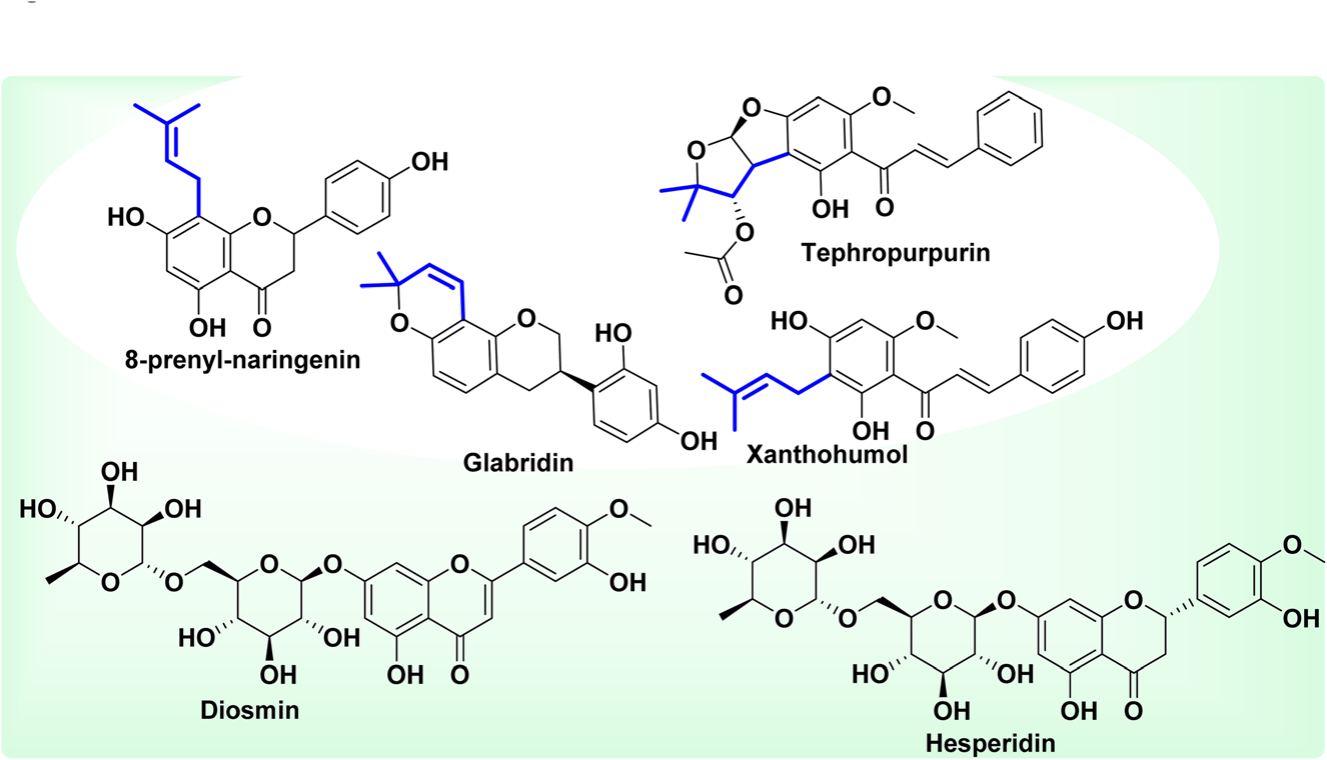
Some flavonoids approved and marketed as drugs or dietary supplements.

The flavonoids diosmin and hesperidin were isolated from *Agathosma betulina*, a South African plant and are also found in *Citrus reticulata* and *Hyssopus officinalis*.^16^ These flavonoids are used in the management of cholera, prostatitis, fever and many other conditions.^48^ These two molecules are active principles of the medicine sold under the name Diosmin Hesperidin® to treat pain and bleeding of haemorrhoids, chronic venous disease and chronic lymphedema. They are also sold as multivitamins and characterized by their high bioavailability.^14–16^ Some of the previous active ingredients from plants are summarized in Fig. 4.

The genus *Erythrina* has been investigated for prenylated flavonoid since 1981 and these investigations are still ongoing. Prenylated flavonoids represent one of the major classes of secondary metabolites emanated from those studies. As mentioned above, a number of prenylated flavonoids have been studied for their health benefits, and these studies resulted in the formulation of several drugs marketed as dietary supplements. Therefore, summarizing previous studies on the prenylated flavonoids content of *Erythrina* plants is of paramount importance. No report had focussed on this subclass of secondary metabolites before from the species *Erythrina*. The intention of this work is to attract the attention of pharmacists/pharmaceutics scientists on *Erythrina* prenylated flavonoids distributed in Africa.

## 3 Extraction and isolation

A total of twenty plant species of the *Erythrina* genus were collected and investigated within African countries (Table 1). It emerges from Table 1 that most chemical studies conducted on the *Erythrina* species have been in Cameroon, followed by Nigeria, Kenya and Botswana.

**Table 1:** African countries where studied *Erythrina* species were collected

Species investigated	Country(ies) of origin and references
*Erythrina caffra*	South Africa^8^
	Botswana^49^
	Egypt^50^
*Erythrina mildbraedii*	Cameroon^51–55^
	Nigeria^56^
*Erythrina vogelii*	Cameroon^57^
	Ivory coast^58^
	Nigeria^59^
*Erythrina sigmoidea*	Cameroon^60–64^
	Nigeria^65–67^
*Erythrina eriotricha*	Cameroon^68^
*Erythrina indica*	Nigeria^59,69^
*Erythrina lysistemon*	Cameroon^70^
	Egypt^71^
	Botswana^72^
*Erythrina sacleuxii*	Kenya^73–76^
*Erythrina addisoniae*	Cameroon^77,78^
	Ghana^79–81^
*Erythrina latissima*	Botswana^82,83^
	South Africa^84^
*Erythrina burana*	Ethiopia^85^
*Erythrina abyssinica*	Botswana^86^
	Kenya^87–89^
	Uganda^90–92^
*Erythrina livingstoniana*	Botswana^93^
*Erythrina brucei*	Ethiopia^94^
*Erythrina burttii*	Kenya^95^
*Erythrina droogmansiana*	Cameroon^96^
	Congo^97^
*Erythrina senegalensis*	Cameroon^98–101^
	Nigeria^102–106^
	Mali^107^
*Erythrina excels*	Cameroon^9,108^
	Kenya^109^
*Erythrina melanacantha*	Kenya^110,111^
*Erythrina schliebenii*	Tanzania^112^

**Fig. 5:**
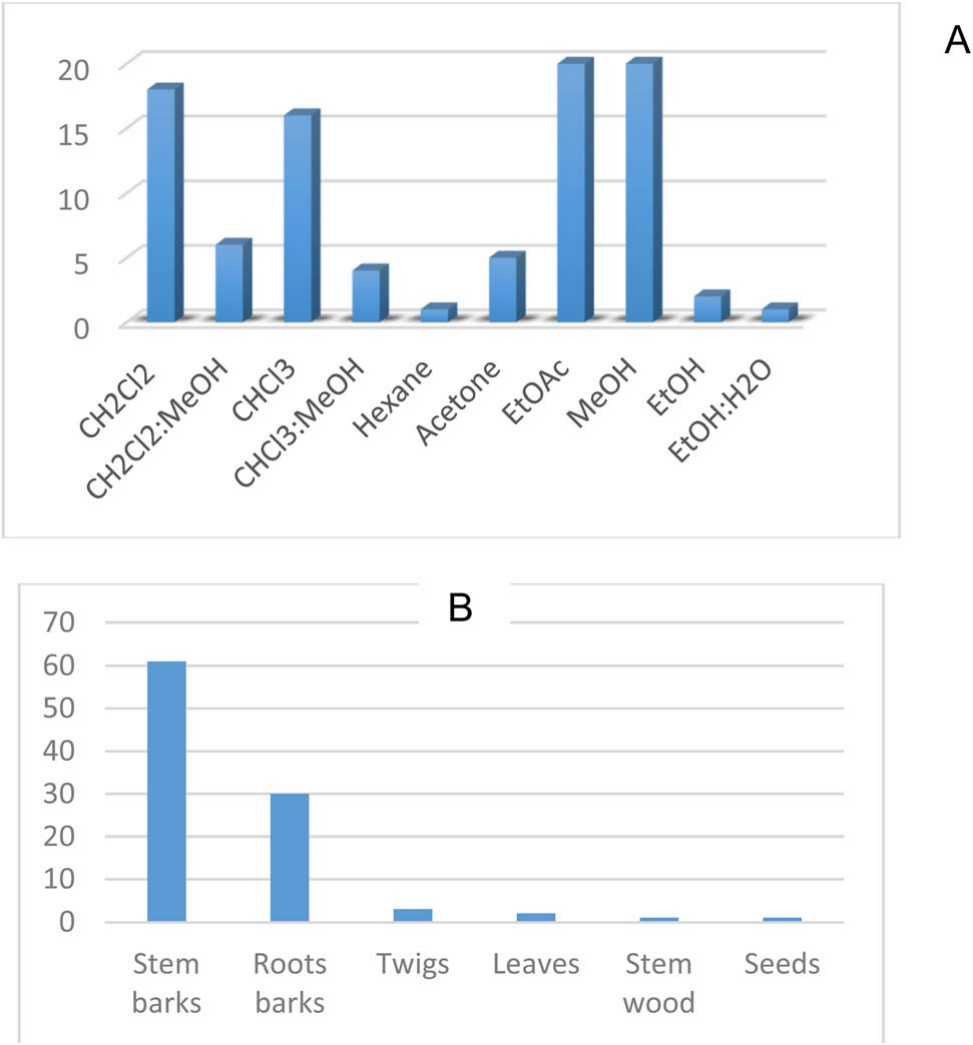
Extracting solvents (A) and main studied plant parts (B) for the plants of *Erythrina* genus.

Maceration appears to have been largely used as an extraction method for the plants reported in this review. Several solvents such as MeOH, EtOH, EtOAc, Acetone, CHCl_3,_ and CH_2_Cl_2_ were used. While generally a single solvent was used, some authors combined two solvents to perform their extraction. The commonly used combinations were CH_2_Cl_2_ : MeOH (1 : 1), and CHCl_3_ : MeOH (1 : 1), EtOH : H_2_O (2 : 1). With regards to the studied plant parts, mostly stem barks were investigated (Fig. 5).

The description follows a specific trend, ranging from prenylated flavanones, flavones, chalcones, isoflavanones, isoflavones, isoflavans, isoflav-3-enes to pterocarpans and pterocarpenes. The structures are also organised in such a way that similar structures within the same subclass are gathered together independently of the species from which they were isolated.

### 3.1 Prenylated flavanones

**Fig. 6:**
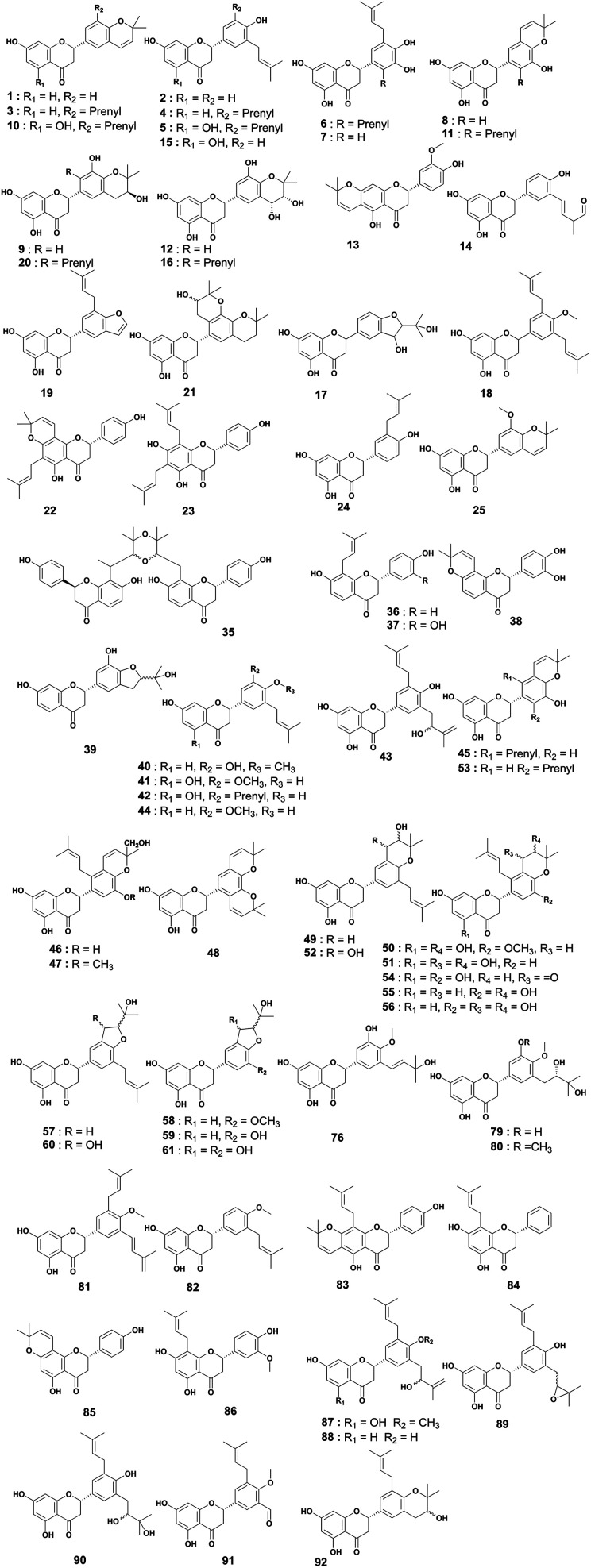
Prenylated flavanones from *Erythrina* plants.

Prenylated flavanones have been largely isolated from *Erythrina* plant species growing in Africa. They represent about 31% of compounds reported herein. The roots of *E. abyssinica* were extracted with MeOH, and chemical investigation of the extract led to the isolation of five flavanones, abyssinone I (1), abyssinone II (2), abyssinone III (3), abyssinone IV (4), and abyssinone V (5).^113^ Three flavanones, namely, sigmoidin A–C (6–8) were obtained from the chloroform extract of *E. sigmoidea* stem bark.^60,64,114^ Further investigation on the same plant materials led to the discovery of sigmoidin D (9).^61^ During continuous studies on the same plant, sigmoidin E (10) and sigmoidin F (11) were also isolated.^62,115^ Sigmoidin G (12), and eriotrinol (13) were reported from the EtOAc extract of *E. sigmoidea* stem bark.^63^ Unfortunately, no configuration was indicated for the stereocenter C-2, but according to Promsattha *et al.* (1989), the C-ring is behind the plane due to the high coupling constant value (*J* = 12.0 Hz).^115^ Furthermore, the hydroxyl groups on the heterocyclic ring possess either (*R*,*S*) or (*S*,*R*) configurations due to the value of the coupling constant reported by the authors. Another two prenylated flavanones, namely, sigmoidin L (14), and 3′-prenylnaringinin (15), were obtained from the EtOAc soluble fraction of *E. sigmoidea* stem bark.^116^ The configuration of the stereocenter was deduced according to the value of the coupling constant (*J* = 13.6 Hz) and the minimal interactions from the chair conformations. A year later, other sigmoidin derivatives, sigmoidins M (16) and N (17), were characterized from the MeOH extract of the stem bark of the same plant.^66^ From the stem bark MeOH extract of *E. abyssinica*, four prenylated flavanones were isolated, with their structures elucidated as abyssinone V 4′-methyl ether (18), abyssinoflavanones IV (19), V (20), and VI (21)^87^ (Fig. 6).

Erythrisenegalone (22) was isolated from the chloroform extract of the stem bark of *E. senegalensis*^105^ while a prenylated flavanone named senegalensein (23) was isolated from the CHCl_3_ extract of the same plant.^102^ Similarly, 3′-prenylnaringenin (24) was isolated from the chloroform extract of the stem bark of *E. eriotricha*.^117^ Ichimaru and coworkers investigated the chemical constituents of the methanol crude extract of the stem bark of *E. abyssinica* and obtained three new prenylated flavanones, namely, abyssinin I (25), II (26), and III (27).^89^ The acetone extract of the stem bark of *E. caffra* Thunb was subjected to chemical studies and yielded burttinone (28).^8^ Two prenylated flavanones, *i.e.*, erycaffra D (29), and erycaffra F (30), were isolated from the EtOAc extract of the stem bark of *E. caffra*.^118^ From the root bark using an EtOAc extract of *E. mildbraedii*, two flavanones were isolated, abyssinone IV-4′-*O*-methyl ether (31) and 7-hydroxy-4′-methoxy-3′-(3-hydroxy-3-methyl-*trans*-but-1-enyl)-5′-(3-methylbut-2-enyl)flavanone (32).^51^ Ali *et al.*, in 2012 reported one previously undescribed prenylated flavanone, trivially named mildbone (33), from the methanol extract of *E. mildbraedii* roots.^54^ The flavanone licoflavanone-4-*O*-methyl ether (34) was obtained from the EtOAc crude extract of the root bark of *E. mildbraedii*.^52^ Structures 26–34 are reported in Fig. S1 (ESI data†).

A biflavanone named *bis*-sigmodiol (35) and a flavanone isobavachin (36) were isolated and characterized from the MeOH extract of the stem bark of *E. sigmoidea*,^119^ in addition to 8-prenyl-7,3′,4′-trihydroxyflavanone (37), sigmone (38) and sigmotriol (39).^67^ Unfortunately, the structure of *bis*-sigmodiol (35) was not fully characterized,^119^ as the configuration of the stereocenter in the prenyl moieties was not determined. However, as the authors declared that the optical rotation was close to zero and used it to support the dimerization, it is possible that it might be the enantiomeric mixture of both monomers. These monomers have been isolated and characterized as brosimacutins A and B.^120^ Erylatissin C (40) was isolated^82^ from the stem wood of *E. latissima*, extracted with the mixture CHCl_3_ : MeOH (1 : 1).^121^ Wanjala and Majinda investigated the CHCl_3_ : MeOH (1 : 1) extract of *E. latissima* stem bark which led to the isolation and characterization of 4′,5,7-trihydroxy-3′-methoxy-5′-prenylflavanone (41) and 4′,5,7-trihydroxy-3′,5′-diprenylflavanone (42).^83^ Two flavanones, named abyssinoflavanone VII (43), and 5-deoxyabyssinin II (44), were obtained from the MeOH extract of the stem bark of *E. abyssinica*^122^ (Fig. 6).

From the methanol extract of the stem bark of *E. abyssinica*, twelve unreported flavanones were characterized, including 2(*S*)-5,5′,7-trihydroxy-2′-prenyl-(2′′,2′′-dimethylpyrano)-(5′′,6′′:3′,4′)flavanone (45), 2(*S*)-5,5′,7-trihydroxy-[2′′-(5′′-hydroxy)-methylpyrano]-(5′′,6′′:3′,4′)flavanone (46), 2(*S*)-5,7-dihydroxy-3′-methoxy-[2′′-(5′′-hydroxy)-methylpyrano]-(5′′,6′′:3′,4′)flavanone (47), 2(*S*)-5,7-dihydroxy-[(5′′,6′′:3′,4′)-(2′′,2′′-dimethylpyrano)-(5′′′,6′′′:5′,6′)]-(2′′′,2′′′-dimethylpyrano)flavanone (48), 2(*S*)-5,7-dihydroxy-5′-prenyl-[2′′,2′′-(3′′-hydroxy)-dimethylpyrano]-(5′′,6′′:3′,4′)flavanone (49), 2(*S*)-5,7-dihydroxy-5′-methoxy-[2′′,2′′-(3′′-hydroxy)-dimethylpyrano]-(5′′,6′′:3′,4′)flavanone (50), 2(*S*)-5,7-dihydroxy-[2′′,2′′-(3′′,4′′-dihydroxy)-dimethylpyrano]-(5′′,6′′:3′,4′)flavanone (51), 2(*S*)-5,7-dihydroxy-5′-prenyl-[2′′,2′′-(3′′,4′′-dihydroxy)-dimethylpyrano)]-(5′′,6′′:3′,4′)flavanone (52), 2(*S*)-5,6′,7-trihydroxy-5′-prenyl-[2′′,2′′-(3′′,4′′-dihydroxy)-dimethylpyrano]-(5′′,6′′:3′,4′)flavanone (53), 2(*S*)-5,5′,7-trihydroxy-[2′′,2′′-(4′′-chromanone)-dimethylpyrano]-(5′′,6′′:3′,4′)flavanone (54), 2(*S*)-5′,7-dihydroxy-[2′′,2′′-(3′′-hydroxy)-dimethylpyrano]-(5′′,6′′:3′,4′)flavanone (55) and 2(*S*)-5′,7-dihydroxy-[2′′,2′′-(3′′,4′′-dihydroxy)-dimethylpyrano]-(5′′,6′′:3′,4′)flavanone (56)^90^ (Fig. 6).

The chemical study of the methanol extract of *E. abyssinica* stem bark led to the isolation and characterization of six unknown flavanones identified as (2*S*)-5,7-dihydroxy-3′-prenyl-2′′-(4′′-hydroxyisopropyl)dihydrofurano[1′′,3′′:4′,5']flavanone (57), (2*S*)-5,7-dihydroxy-3′-methoxy-2′′-(4′′-hydroxyisopropyl)dihydrofurano[1′′,3′′:4′,5']flavanone (58), (2*S*)-5,7,3′-trihydroxy-2′′-(4′′-hydroxyisopropyl)dihydrofurano[1′′,3′′:4′,5']flavanone (59), (2*S*)-5,7-dihydroxy-3′-prenyl-3′′-hydroxy-dihydrofurano[1′′,3′′:4′,5']flavanone (60), (2*S*)-5,7,3′-trihydroxy-2′′-(4′′-hydroxyisopropyl)-3′′-hydroxy-hydrofurano[1′′,3′′:4′,5']flavanone (61) and (2*S*)-5,7,3′-trihydroxy-2′-prenyl-2′′-(4′′-hydroxyisopropyl)-3′′-hydroxy-dihydrofurano[1′′,3′′:4′,5']flavanone (62).^91^ A total of nine prenylated flavonoids, namely, erylatissins D, E, G (63–65), dihydroabyssinin I (66), 3′4′-dihydro-3′-hydroxy-8′-methylether of sigmoidin C (67), 4′-*O*-methylsigmoidin B (68), abyssinoflavone IV (69) and V (70), were obtained from the crude MeOH extract of *E. latissima* stem bark.^84^ The chemical investigation of the CHCl_3_ : MeOH (1 : 1) extract of twigs and roots of *E. abyssinica* resulted in the isolation of one unknown flavanone, abyssinone VII (71).^86^ From the EtOAc crude extract of *E. abyssinica* root bark, several compounds, including 7-hydroxy-2-[4-methoxy-3-(3-methylbut-2-enyl)phenyl]chroman-4-one (72), erythribyssin G (73) and erythribyssin I (74)^123^ were reported. The EtOAc extract of the stem bark of *E. livingstoniana* was chemically investigated and led to the isolation of four prenylated flavanones, erylivingstone B (75), erylivingstone C (76), 5,7-dihydroxy-3′,4′-dimethoxy-5′-prenylflavanone (77) and 7,3′-dihydroxy-4′-methoxy-5′-prenylflavanone (78).^93^ Additionally, these authors investigated the CH_2_Cl_2_ : MeOH (1 : 1) extract of the twigs of the same plant and characterized two new flavanones, namely, 5,7,3′-trihydroxy-5′-(2,3-dihydroxy-3-methylbutyl)-4′-methoxy flavanone (79) and 5,7-dihydroxy-5′-(2,3-dihydroxy-3-methylbutyl)-3′,4′-dimethoxyflavanone (80).^121^ An investigation of the acetone extract of the stem bark of *E. burttii* led to the isolation of burttinonedehydrate (81).^124^ 4′-Methoxylicoflavanone (82) was isolated from the EtOAc extract of roots bark of *E. droogmansiana*,^125^ while lupinifolin (83) was obtained from the combined hexane and CH_2_Cl_2_ extracts of the stem bark of *E. excelsa*.^109^ Three prenylated flavanones were isolated from the CH_2_Cl_2_ extract of stem bark from *E. melanacantha* ssp. melanacantha Taub. ex Harms, yielding glabranin (84), citflavanone (85) and exiguaflavanone (86).^110^ The chemical study of the CH_2_Cl_2_ extract of *E. addisoniae* stem bark led to the isolation of two flavanones, namely 2*S*-3′-(2-hydroxy-3-methylbut-3-enyl)licoflavone-4′-methyl ether (87) and 2*S*-3′-(2-hydroxy-3-methylbut-3-enyl)abyssinone II (88).^80^ Four flavanones were reported from the CH_2_Cl_2_ extract of the stem bark of *E. addisoniae*. These compounds included addisoniaflavanone I (89), II (90), and III (91) as well as 5,7-dihydroxy-5′-prenyl-[2′′,2′′-(3′′-hydroxy)-dimethylpyrano]-(5′′,6′′:3′,4′)flavanone (92)^81^ (Fig. 6). Structures 65–75 are found in Fig. S1 (ESI†).

### 3.2 Prenylated flavones

**Fig. 7:**
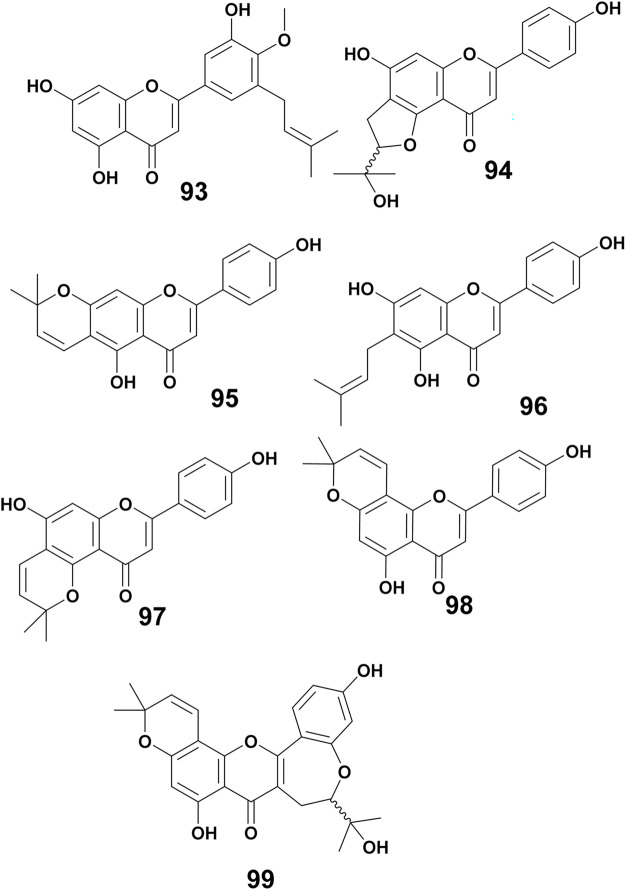
Prenylated flavones from *Erythrina* plants.

From the crude MeOH extract of the stem bark of *E. latissima* 5′-prenylpratensein A (93) was characterized (Fig. 6).^84^ The chemical study of the ethanol leaf extract of the medicinal plant *E. vogelii* led to the isolation and characterization of one unreported flavone, vogeol (94), as well as known compounds carpachromene (95) and 5,7,4′-trihydroxy-6-prenylflavone (96).^57^ One new flavone, vogelin J (97), was characterized from the CH_2_Cl_2_ : MeOH (1 : 1) extract of the stem bark of *E. vogelii*.^126^ The study of the MeOH extract of *E. sigmoidea* stem bark afforded atalantoflavone/limonianin (98) and neocyclomorusin (99)^99^ (Fig. 7).

### 3.3 Prenylated chalcones

**Fig. 8:**
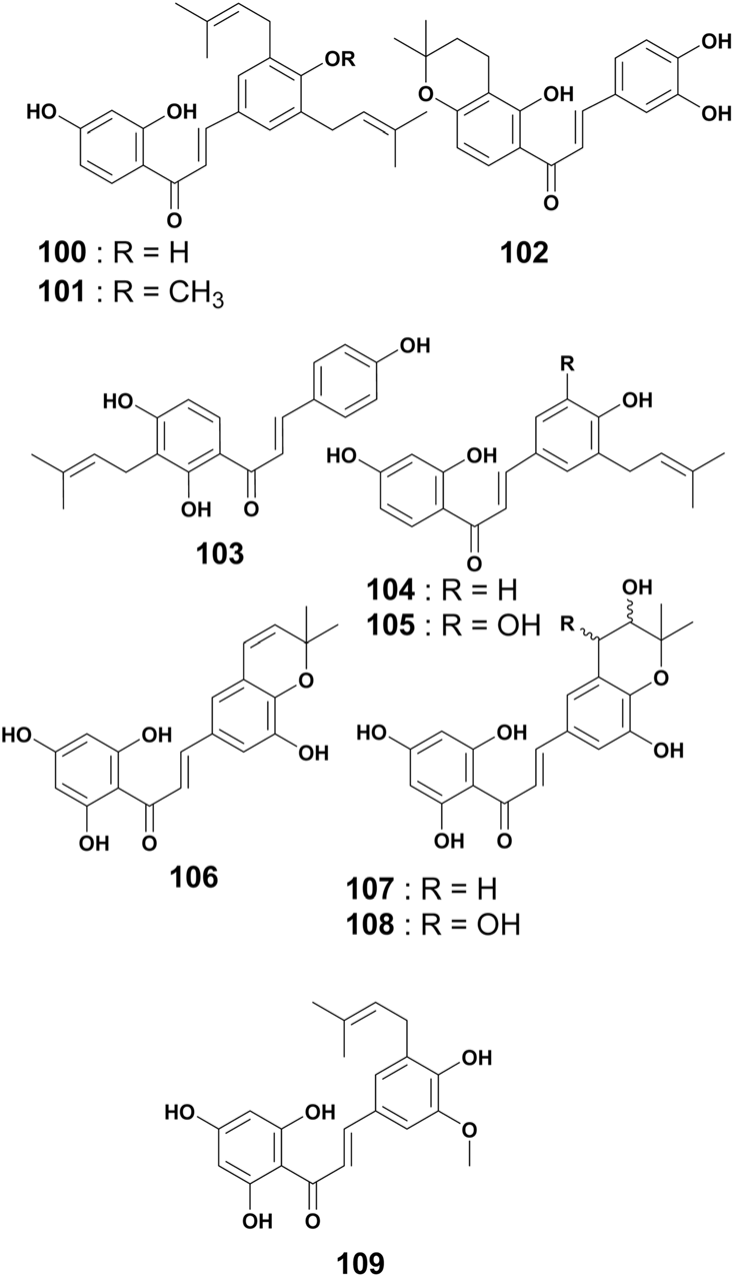
Prenylated chalcones from *Erythrina* plants.

The chemical investigation of the MeOH extract of root bark of *E. abyssinica* led to the isolation of abyssinone VI (100).^113^ From the EtOAc extract of the root bark of *E. mildbraedii*, one chalcone named abyssinone VI-4′-*O*-methyl ether (101) was obtained.^51^ In addition, mildbenone (102) was reported from the methanol extract of the roots of the same plant.^54^ Isobavachalcone (103) was isolated from the CHCl_3_ : MeOH (1 : 1) extract of the stem wood of *E. latissima*.^82^ Licoagrochalcone A (104) is a prenylated chalcone obtained from the CHCl_3_ : MeOH (1 : 1) extract of twigs and roots of *E. abyssinica*.^86^ Compound 5-prenylbutein (105) was isolated from the EtOAc extract of the stem bark of the same plant.^127^ Further investigation on the MeOH extract of stem bark of the same plant also led to the discovery of four chalcones, *i.e.*, abyssinone A–D (106–109)^128^ (Fig. 8).

### 3.4 Prenylated isoflavanones

**Fig. 9:**
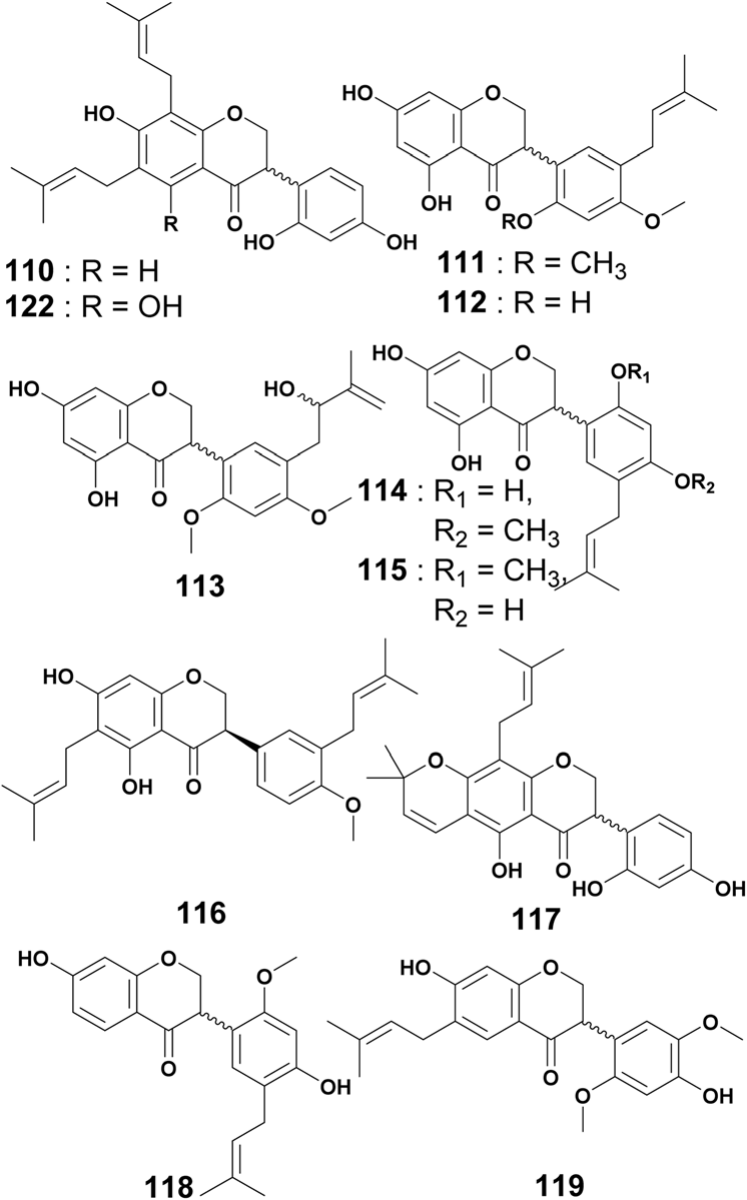
Prenylated isoflavanones from *Erythrina* plants.

The isoflavanone eriotrichin B (110) was reported from the CH_2_Cl_2_ extract of the root bark of *E. eriotricha*. Its configuration at C-3 was not determined.^129^ The chemical investigation of the EtOAc extract of the stem bark of *E. caffra* led to the isolation of erycaffra A (111), erycaffra B (112) and lysisteisoflavanone (113) (configurations at C-3 were not determined).^49,130^ The chemical study of the CH_2_Cl_2_ extract of the roots of *E. vogelii* yielded two isoflavanones identified as vogelins A (114) and B (115).^58^ Another derivative, Vogelin D (116) was discovered from the CH_2_Cl_2_ crude extract of the root bark of the same plant.^131^ Another prenylated isoflavanone is 2,3-dihydroauriculatin (117) characterized from the CH_2_Cl_2_ : MeOH (1 : 1) extract of the stem bark of *E. vogelii*.^126^ One isoflavanone, sigmoidin I (118) was reported from the MeOH crude extract of *E. sigmoidea* root bark,^129^ as well as its congener named sigmoidin J (119).^132^ (R)-saclenone (120), was obtained from the CH_2_Cl_2_ extract of the stem bark of *E. sacleuxii*.^74^ The investigation of the methanol extract of the stem bark of *E. addisoniae* led to the discovery of 5,2′,4′-trihydroxy-6-(γ,γ-dimethylallyl)-2′′′,2′′′-dimethyldihydropyrano[5′′′,6′′′]isoflavone/hydroxyosajin (121) and orientanol E (122).^77^ Unfortunately, the configuration of the stereogenic center of 122 was not indicated since its first isolation.^133^ This underscores the needs for continuous investigations on the *Erythrina species* to close some gaps encountered in the literature. The study of the chloroform extract of the leaves of *E. lysistemon* afforded 2′,5,7-trihydroxy-4-methoxy-5-prenylisoflavanone (123).^72^ The MeOH crude extract of the root bark of *E. addisoniae* afforded erythraddisons III and IV (124, 125).^78^ From the EtOAc crude extract of the root bark of *E. abyssinica*, erythribyssin E (126), erythribyssin J (127) and prostratol C (128), were isolated.^123^ The stem bark extract of *E. brucei* led to the characterization of bruceins A (129) and B (130), along with kenusanone F (131), 7-methylkenusanone F (132), and sophoraisoflavanone A (133).^94^ Two isoflavanones, erydroogmansin A (134) and erypoegin D (135), were isolated from the CH_2_Cl_2_ : MeOH (1 : 1) extract of the root bark of *E. droogmansiana*.^97^ Sigmoidin H (136) was isolated from the CH_2_Cl_2_ : MeOH (1 : 1) extract of the roots of *E. senegalensis*.^108^ The phytochemical investigation of the MeOH extract of the same plant led to the isolation of two new isoflavanones, erysenegalenseins B (137) and C (138) (Fig. 9). Unfortunately, the configurations of stereocenters of those compounds were not indicated.^134^ Structures 120–138 are incorporated in Fig. S1 (ESI†).

### 3.5 Prenylated isoflavones

**Fig. 10:**
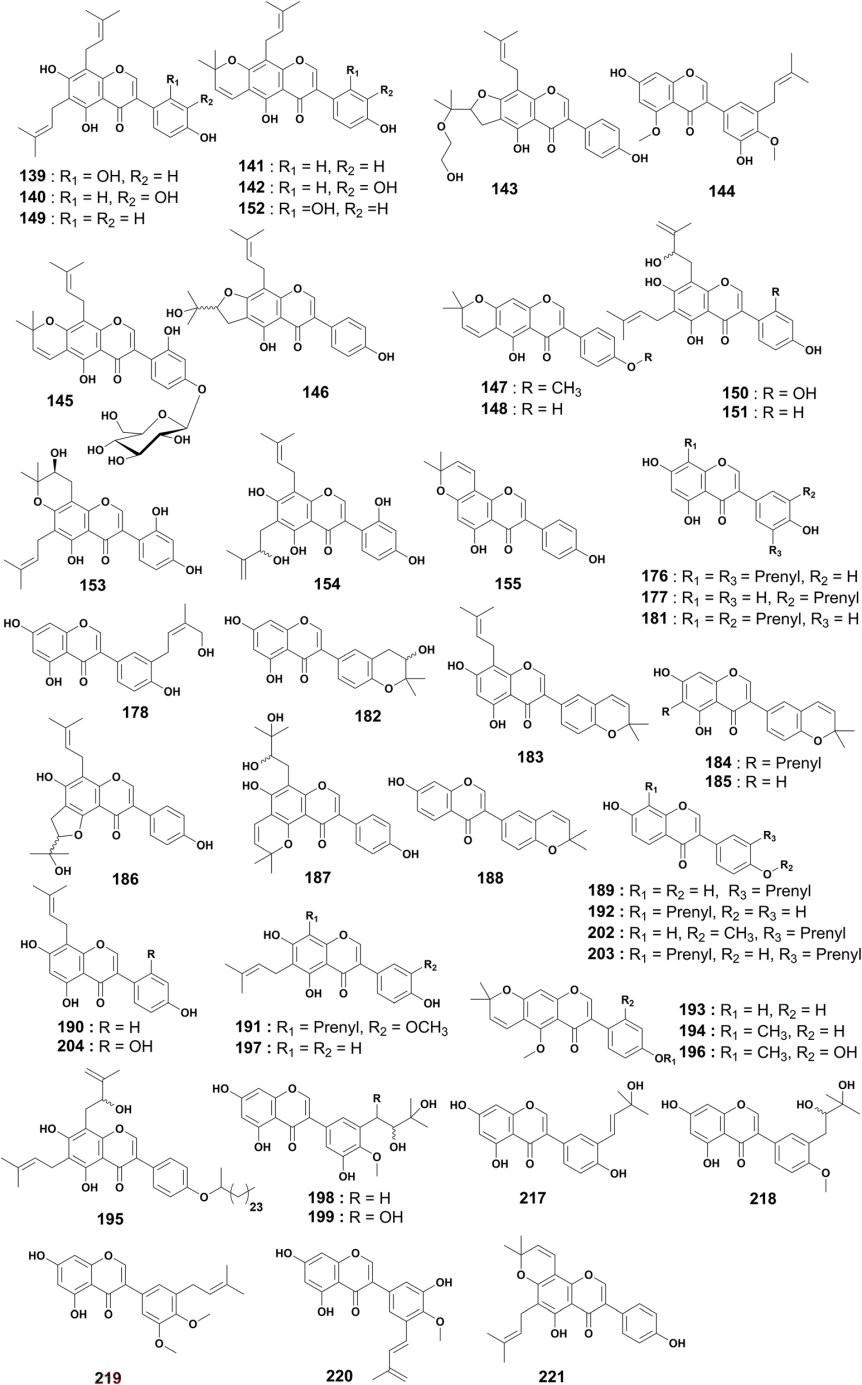
Prenylated isoflavones from *Erythrina* plants.

This subclass represents 28% of prenylated flavonoids derived from *Erythrina* species distributed throughout Africa. The isoflavones 8-prenylluteone (139), 6,8-diprenylorobol (140), scandenone (141) and auriculasin (142) were isolated from the chloroform extract of the stem barks of *E. eriotricha*.^135^ Eriotriochin (143) was obtained from the chloroform extract of the stem bark of the same plant.^136^ Further investigations on the same species led to the isolation of 5,4′-dimethoxy-3′-prenylbiochanin A (144).^137^ The first glycosylated prenyl isoflavone auriculatin 4′-*O*-glucoside (145) and 8-prenylerythrinin C (146) were characterized from the chloroform extract of the stem bark of *E. eriotricha*.^138^ 4′-*O*-Methylalpinumisoflavone (147) is one of the secondary metabolites reported from the EtOAc extract of *E. sigmoidea* stem bark.^63^ The acetone extract of the stem bark of *E. caffra* afforded two isoflavones, alpinumisoflavone (148) and 6,8-diprenylgenistein (149).^8^ Erysenegalensein D (150) and erysenegalensein E (151) are prenylated isoflavones obtained from the MeOH extract of the stem bark of *E. senegalensis*.^139^ The chemical study of the CH_2_Cl_2_ crude extract of the stem bark of the same plant led to the characterization of four prenylated isoflavones named auriculatin (152), erysenegalensein O (153), erysenegalensein N (154) and derrone (155)^100,101^ (Fig. 10).

Two epoxy isoflavones reported as erysenegalensein F (156) and erysenegalensein G (157) were isolated from the MeOH extract of *E. senegalensis* stem bark.^140^ Erysenegalensein L (158) and erysenegalensein M (159) were obtained from the MeOH extract of seeds of the same plant.^139^ Erycaffra C (160), isoerysenegalensein E (161), isosenegalensein (162), erythrinin C (163) and erysubin B (164) were obtained from the stem bark EtOAc extract of *E. caffra*.^49,87^ Another study was later conducted by the same authors on the EtOAc extract of stem bark of *E. caffra* to yield laburnetin (165).^130^ Tchokouaha and coworkers reported several prenylated isoflavones from the dichloromethane extract of the stem bark of *E. mildbraedii*. These compounds comprise two previously unreported derivatives named erymildbraedins A (166) and B (167) (Fig. S1, ESI†) as well as 5,4′-dihydroxy-2′-methoxy-8-(3,3-dimethylallyl)-2′′,2′′dimethylpyrano[5,6 : 6,7]isoflvone (168) and eryvarin B (169).^53^ Three isoflavones 2′,7-dihydroxy-4-methoxy-5′-prenylisoflavone (170), erythrinin B (171), and parvisoflavone B (172), were reported from the EtOAc crude extract of *E. mildbraedii* root bark.^52^ The chemical study of the ethanol extract of the leaves of *E. vogelii* led to the isolation of vogeliiol (173), euchrenone b_10_ (174) and 5,4′-dihydroxy-8-(3′′-methylbut-2′′-enyl)-2′′′-(4′′′-hydroxy-4′′′-methylethyl)-furano-[4′′′,5′′′:6,7] isoflavone (175)^57^ (Fig. S1, ESI†).

From the roots of *E. vogelii*, two isoflavones were isolated with their structures being elucidated as vogelin C (176) and isowighteone (177).^58^ Three unreported isoflavones, vogelins E (178), F (179) and G (180), in addition to five reported isoflavones, namely isolupalbigenin (181), ficuisoflavone (182), ulexone A (183), isochandalon (184) and isoderrone (185) were obtained from the CH_2_Cl_2_ crude extract of *E. vogelii* root bark.^131^ Two new isoflavones named vogelins H (186) and I (187) were characterized from the CH_2_Cl_2_ : MeOH (1 : 1) extract of stem bark of the same plant.^126^ Corylin (188), and neobavaisoflavone (189) were isolated from the MeOH crude extract of *E. sigmoidea* root bark.^129^ One isoflavone, lupiwighteone (190), was isolated from the MeOH extract of the stem bark of the above mentioned plant.^119^ Two isoflavones, fleminphilippinin B (191) and 8-prenyldaidzein (192), were isolated from the EtOAc extract of *E. sigmoidea* stem bark.^68^ Two isoflavones, indicanine C (193) and 5,4′-di-*O*-methylalpinumisoflavone (194) were reported from the CH_2_Cl_2_ : MeOH extract of the roots bark of *E. indica*.^59^ The novel isoflavones indicanines D (195) and E (196) along with wighteone (197) were isolated from the CH_2_Cl_2_ : MeOH (1 : 1) extract of the stem bark of *E. indica*. Compound 195 featured an alkane chain containing 26 carbon atoms attached to the hydroxyl at position 4′.^69^ Four isoflavones erysacleuxins A (198) and B (199), 5′-prenylpratensein (200), and 3′-prenylbiochanin A (201) were reported from both the chloroform and EtOAc extracts of *E. sacleuxii* stem bark.^73,76^ The unreported isoflavonoid 5-deoxy-3′-prenylbiochanin A (202) along with the known erysubin F (203) were isolated and characterized from the acetone extract of the root bark of *E. sacleuxii*.^75^ One isoflavone, 2,3-dehydrokievitone (204), was obtained from the CH_2_Cl_2_ extract of the stem bark of the same plant^74^ (Fig. 10).

The chemical study of the methanol stem bark extract of *E. addisoniae* led to the isolation of warangalone 4′-methyl ether (205).^77^ The EtOAc extract of the twigs of *E. lysistemon* yielded 4′,7-dihydroxy-2′′,2′′-dimethylpyrano [5′′,6'':5,6]-isoflavone (206) and 4′,5,7-trihydroxy-6-(2′′-hydroxy-3′′-prenyl)isoflavone (207).^72^ The MeOH crude extract of the root bark of *E. addisoniae* led to the isolation of erythraddisons I (208) and II (209).^78^ Erylatissin F (210) and glycyrrhizoflavone (211) were isolated from the crude MeOH extract of *E. latissima* stem bark.^84^ The chemical investigation of the CHCl_3_ : MeOH (1 : 1) extract of the twigs and roots of *E. abyssinica* resulted in the identification of semilicoisoflavone B (212).^86^ However, 212 might also result from the direct Claysen cyclization of the prenyl group at C-3′ and the hydroxyl at C-4′ in 211. The investigation of the acetone extract of the stem bark of *E. burttii* yielded 7-*O*-methylluteone (213).^124^ The isoflavone erydroogmansin B (214) was isolated from the CH_2_Cl_2_ : MeOH (1 : 1) extract of the root bark of *E. droogmansiana*.^97^ Excelsanone (215) was obtained from the EtOH : H_2_O (8 : 2) extract of the stem bark of *E. excelsa*.^9^

The chemical study of the MeOH extract of the stem bark of *E. schliebenii* led to the isolation and characterization of schliebenones A (216) and C (217), 5,7-dihydroxy-4′-methoxy-3′-(2,3-dihydroxy-3-methylbutyl)isoflavone (218) and 5′-methoxy-3′-prenylbiochanin A or piscerythrinetin (219), while schliebenone B (220) is a secondary metabolite of the MeOH extract of the root bark of this plant.^112^ Osajin (221) was isolated from the EtOH : H_2_O (3 : 2) extract of the stem bark of *E. senegalensis*^106^ (Fig. 10). Structures 205–216 are shown in Fig. S1 (ESI†).

### 3.6 Prenylated isoflavanes and isoflav-3-enes

**Fig. 11:**
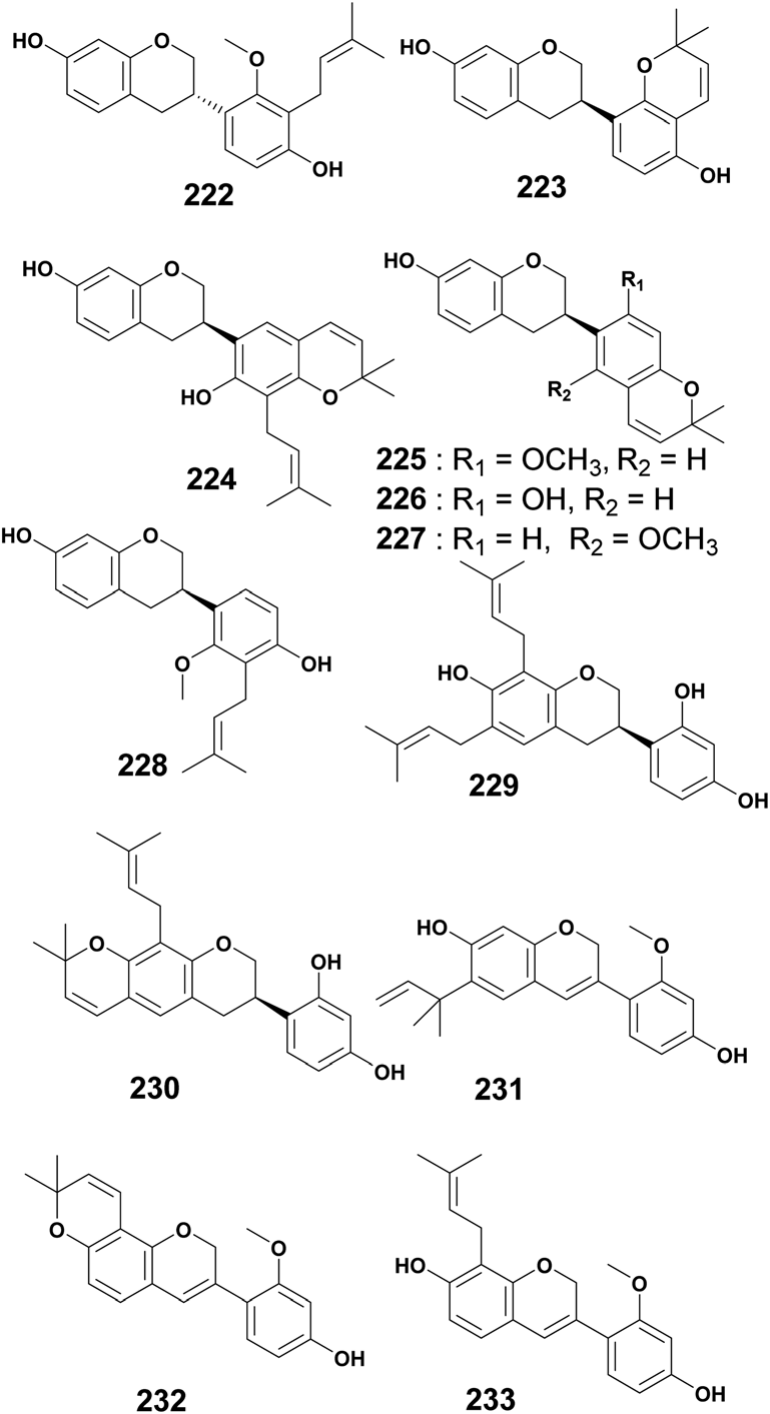
Prenylated isoflavanes and isoflav-3-enes from *Erythrina* plants.

El-Masry *et al.* (2010) reported the isolation and characterization of two prenylated isoflavanes, 3*S* (+) 2′-*O*-methylphaseollidinisoflavan (222) and 3*R* (−) erythbidin A (223) from the ethanol extract of the stem bark of *E. caffra*.^50^ The isoflavane (3*R*)-2,7-dihydroxy-3-prenyl-2,2-dimethylpyrano[5,6:4,5]isoflavan (224) was isolated from the EtOAc crude extract of *E. mildbraedii* root bark.^52^ Studies of the CH_2_Cl_2_ : MeOH (1 : 1) extract of the root bark of *E. livingstoniana* led to the characterization of 7-hydroxy-2′-methoxy-[6′′,6′′-dimethylpyrano (2′′,3′′:4′,5′)]isoflavan (225), 7,2′-dihydroxy-[6′′,6′′-dimethyl pyrano (2′′,3′′:4′,5′)]isoflavan (226), 3*R* 2′-methoxyphaseollinisoflavan (227), and 7,4′-dihydroxy-2′-methoxy-3'-(3-methylbut-2-enyl) isoflavan (228).^141^ The chemical study of the CH_2_Cl_2_ root bark extract of *E. lysistemon* led to the isolation of two isoflavans, namely, eryzerin C (229), and eryvarin C (230).^142^ The acetone extract of the root bark of *E. burttii* Ball.f. were investigated and resulted in three isoflav-3-enes simply named burttinol A–C (231–233)^143^ (Fig. 11).

### 3.7 Prenylated pterocarpans and pterocarpenes

**Fig. 12:**
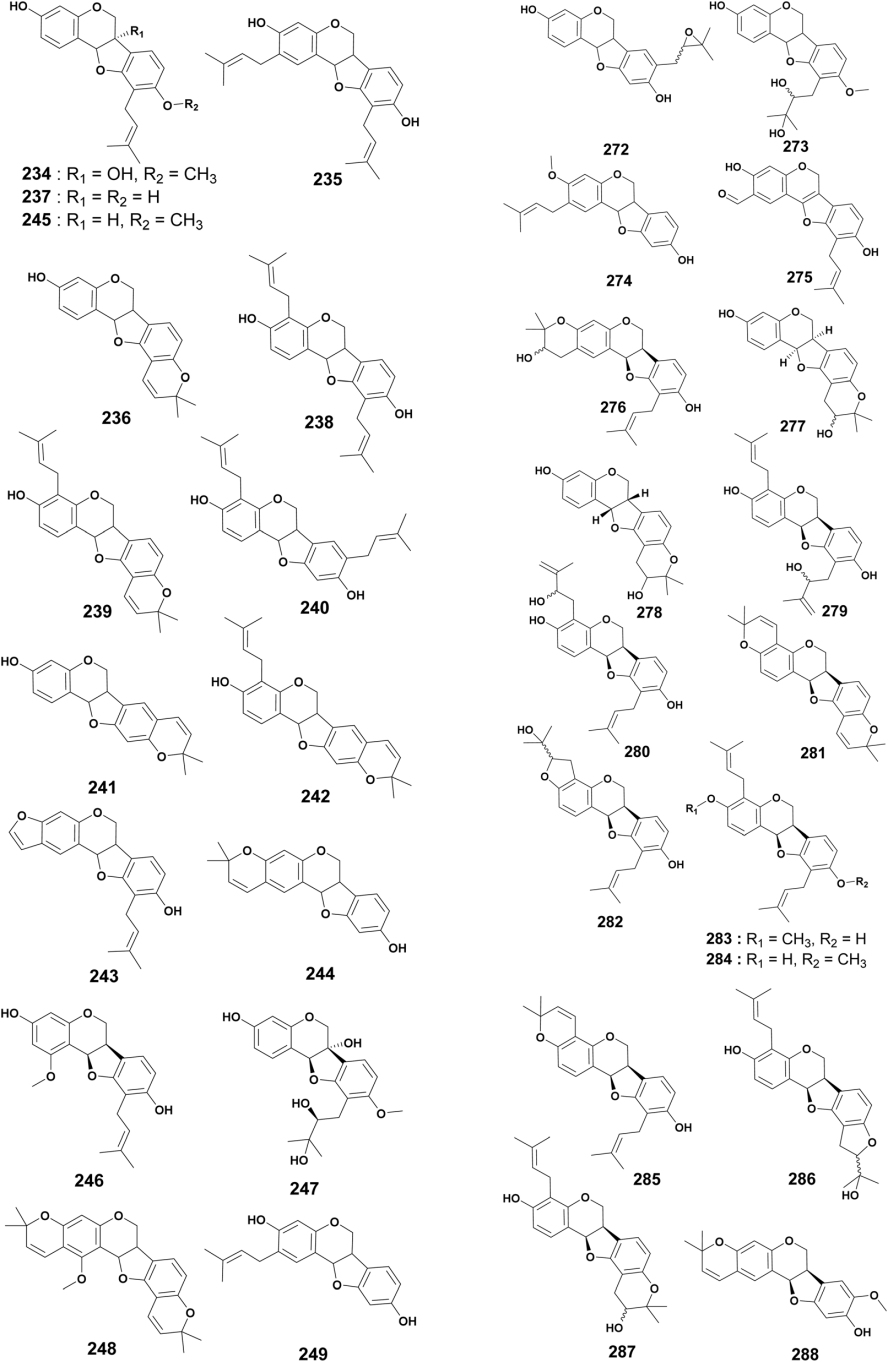
Prenylated pterocarpans from E*rythrina* plants.

As this represents the third largest group of prenylated flavonoids, this section presents the different prenylated pterocarpans. The roots of *E. abyssinica* were extracted with methanol, and the study of its chemical contents revealed the isolation of erythrabyssin I, also named cristacarpin (234), erythrabyssin II (235), phaseollin (236) and phaseollidin (237).^113^ Three new pterocarpans, erybraedins A–C (238–240) and the known isoneorautenol (241) were isolated from the ethanol extract of *E. mildbraedii* roots.^56^ Erybraedins D (242) and E (243) were reported from the MeOH extract of the stem bark of *E. eriotricha*.^144^ The continuous search of bioactive flavonoids led to the isolation of neorautenol (244).^132^ El-Masry and coworkers reported one pterocarpan, sandwicensin (3-hydroxy-10-dimethylallyl-9-methoxypterocarpan) (245) from the ethanol extract of *E. caffra*.^50^ Compound 1-methoxyphaseollidin (246) was isolated from the CH_2_Cl_2_ root extract of *E. vogelii*.^58^ A pterocarpan orientanol A (247) was discovered in the MeOH extract of the stem bark of *E. sigmoidea*.^119^ Two pterocarpans, gangetinin (248) and calopocarpin (249) were isolated from the EtOAc extract of *E. sigmoidea* stem bark^68^ (Fig. 12).

From the methanol extract of the stem bark of *E. lysistemon*, three novel pterocarpans erylysin A–C (250–252) were reported, conjointly with known orientanol C (253), erysubin D (254), eryvarin D (255) and erystagallin C (256).^70^ Shinpterocarpin (257) was isolated from the CH_2_Cl_2_ extract of the stem bark of *E. sacleuxii*.^74^

Sophorapterocarpan A (258), and 6α-hydroxyphaseollidin (259) were isolated and characterized from the MeOH extract of *E. sigmoidea* stem barks.^99^ Nguyen and coworkers studied the chemical composition of the MeOH extract of *E. abyssinica*. They identified two new prenylated pterocarpans, erythribyssin A (260) and C (261), along with five known derivatives identified as eryvarin K (262), 3,9-dihydroxy-4-prenyl-[6a*R*:11 a*R*]pterocarpan (263), folitenol (264), erysubin E (265) and erystagallin A (266).^145^ The stem and root bark of *E. brucei* were extracted with CH_2_Cl_2_ : MeOH (1 : 1) and further chemical investigations resulted in the isolation of eryvarin J (267), 2-prenyl-6α-hydroxyphaseollidin (268), and erycristagallin (269).^94^ The acetone extract of the stem bark of *E. burttii* led to the isolation of 3-*O*-methylcalopocarpin (270).^124^ Erybraedin F (271) was isolated from the CH_2_Cl_2_ extract of the stem bark of *E. senegalensis*.^107^ The chemical structures 250–271 are reported in Fig. S1 (ESI†).

Compound 2′,3′-epoxyhomoedudiol (3-hydroxy-8-(3,3-dimethyl-oxiranylmethyl)pterocarpane (272) was isolated from the CH_2_Cl_2_ extract of the stem bark of *E. melanacantha*.^111^ The chemical study of the CH_2_Cl_2_ root bark extract of *E. schliebenii* led to the isolation and characterization of 3-hydroxy-10-(2,3-dihydroxy-3-methylbutyl)-9-methoxypterocarpan (273) and orientanol B (274).^112^ Erythribyssin O (275), erythribyssin L (276), erythribyssin D (277) and erythribyssin M (278) were isolated from the EtOAc extract of the stem bark of *E. abyssinica*.^146^ The chemical study of the CH_2_Cl_2_ root bark extract of *E. lysistemon* led to the isolation of nine unreported pterocarpans, namely, (6a*R*,11a*R*)-3,9-dihydroxy-4-(γ,γ-dimethylallyl)-10-(2′′-hydroxy-3′′-methylbut-3-enyl) pterocarpan (279), (6a*R*,11a*R*)-3,9-dihydroxy-10-(γ,γ-dimethylallyl)-4-(2′′-hydroxy-3′′-methylbut-3-enyl)pterocarpan (280), (6a*R*,11a*R*)-2′,2′-dimethylpyrano[6′,5′:3,4]-2′′,2′′ dimethylpyrano[6′′,5′′:9,10]pterocarpan (281), (6a*R*,11a*R*)-3,9-dihydroxy-10-(γ,γ-dimethylallyl)-2′-hydroxyisopropyl dihydrofurano[5′,6′:3,4]pterocarpan (282), (6a*R*,11a*R*)-3-methoxy-9-hydroxy-4,l0-di(γ,γ-dimethylallyl)-pterocarpan (283), (6a*R*,11a*R*)-3-Hydroxy-9-methoxy-4,10-di(γ,γ-dimethylallyl)-pterocarpan (284), (6a*R*,11a*R*)-9-hydroxy-10-(γ,γ-dimethylallyl)-2′,2′-dimethylpyrano[6′,5′:3,4]pterocarpan (285), (6a*R*,11a*R*)-3,9-dihydroxy-4-(γ,γ-dimethylallyl)-2′′-hydroxyisopropyl dihydrofurano[5′′,6′′:9,10]pterocarpan (286) and (6a*R*,11a*R*)3-hydroxy-4(γ,γ-dimethylallyl)-2′,2′-(3′′-hydroxy)-dimethylpyrano[6′′,5′′:9,10]pterocarpan (287), as well as the reported 8-methoxyneorautenol (288).^142^ The pterocarpen sigmoidin K (289) was obtained from the MeOH crude extract of *E. sigmoidea* root bark^132^ (Fig. 12).

In these studies of prenylation of flavones from African *Erythrina*, flavanones tend to orient on the B ring cycle, in comparison to isoflavonoids where the prenylation was oriented on both the A and B rings. This preference in flavone and flavanone might be related to high enzyme AnaPT content in the corresponding plants.^26^

## 4 Distribution of prenylated flavonoids in African *Erythrina*

Considerable chemical studies have been conducted on the plant species of *Erythrina* genus (Fabaceae) across the world. Data from previous studies lend to the theory that plants of this genus produce mainly flavonoids^10–12^ and alkaloids.^147,148^ Prenylated flavonoids are relatively dominant in the genus and this section discusses the occurrence of different subclasses of prenylated flavonoids already reported. Some structural differences have often been noted in compounds isolated from the same genus according to geographical location.^149^ In ess ence, there is no difference between the skeletons of prenylated flavonoids occurring in African *Erythrina* species and others worldwide. Focusing the review on African *Erythrina* is only for the purpose of collecting information regarding studies already published. Several structures reported in this review have been equally reported in species growing in Japan, Brazil and India.^150–154^ This section therefore collates the information to support the occurrence of prenylated flavonoids within the genus *Erythrina*. This genus belongs to the Fabaceae family. While the comparison of prenylated flavonoids occurring within the *Erythrina* genus across the world was not the objective of the present review, during the investigation we did notice the occurrence of isoflavonoids and pterocarpans in the species growing in Asia.^150–152,155–157^ There is no review differentiating these compounds from species of other continents, and further reviews might focus on this. A review by Wei *et al.* focussed on all prenylated flavonoids in plants kingdom world-wide, but this review only mentioned ten species of *Erythrina* with prenylated flavonoids.^13^ Another review targeting therapeutic prenylated flavonoids from the Fabaceae family did not provide enough information on the *Erythrina* genus, particularly the species from Africa.^158^ In the present review, the phytochemistry of twenty species of *Erythrina* occurring only in Africa is discussed. We limited our investigation only to those species of the genus *Erythrina* in Africa.

**Fig. 13:**
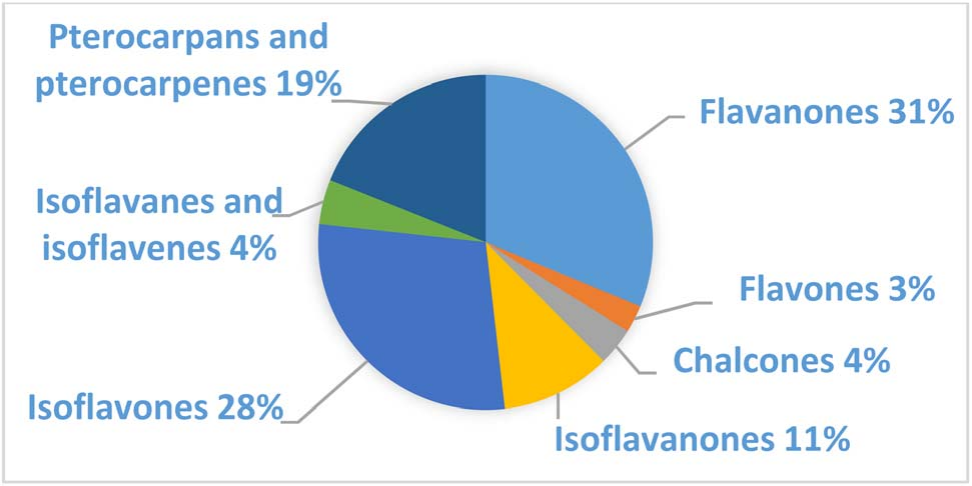
Subclasses of prenylated flavonoids from African *Erythrina*.

Prenylated flavanones represent the higher percentage (31%) of prenylated flavonoids in African *Erythrina*, followed by isoflavones (28%) and pterocarpans (19%) (Fig. 13). Within these three subclasses, certain specific compounds have been isolated several times within the genus.

From this summary of prenylated flavonoids isolated and characterized from the plants of the genus *Erythrina* it appears that abyssinone V (5), sigmoidin A (6), abyssinone V-4′-*O*-methyl ether (18), warangalone (141), 6,8-diprenylgenistein (149) and phaseollidin (237) can be considered to be their chemical markers. Concerning the subclass of prenylated flavanones, abyssinone V (5) and abyssinone V-4′-*O*-methyl ether (18) specifically have been reported from more than ten chemical studies within the genus. According to these reported data, twenty-five isoflavones have been isolated and characterized from more than one *Erythrina* species. Warangalone/scandenone (141), alpinumisoflavone (148), 6,8-diprenylgenistein (149), erysenegalensein E (151), auriculatin (152), neobavaisoflavone (189) and 5′-prenylpratensein (200) are reported here as the chemical markers of the prenylated isoflavones in the genus *Erythrina* growing in Africa. Specifically, warangalone/scandenone (141), and 6,8-diprenylgenistein (149) were characterized from ten phytochemical investigations in the genus. These isoflavones are largely distributed in the stem barks of various *Erythrina* species. As noted from these isolations, pterocarpans represent 19% of compounds isolated from the genus *Erythrina*, with 20 compounds identified in more than one *Erythrina* species. It emerges from this study that erythrabyssin II (235), phaseollin (236), phaseollidin (237), erybraedin A (238), isoneorautenol (241), cristacarpin (234) and calopocarpin (249) are the chemical markers of *Erythrina* genus in Africa for the prenylated pterocarpans, with phaseollidin (237) reported in eleven chemical studies. Regarding the location in erythrina plant parts, in general root bark extracts yielded pterocarpans (Table 2). Pterocarpans were mainly isolated from *E. abyssinica*, *E. senegalensis*, *E. burttii* and *E. eriotricha*, however, these species are distributed across Cameroon, Nigeria, Mali, Kenya and Botswana, and unfortunately this not indicate any specificity regarding their occurrence. The same analysis was deduced from flavanones and isoflavones.

**Table 2:** Distribution of prenylated flavonoids in *Erythrina* genus

Isolated compounds	Species (part studied)	Extract and references
Abyssinone II (2)	*E. latissima* (stem wood)	CHCl_3_ : MeOH (1 : 1)^82,141^
	*E. abyssinica* (root bark)	EtOAc^123^
Abyssinone III (3)	*E. abyssinica* (root bark)	EtOAc^123^
Abyssinone IV (4)	*E. sigmoidea* (stem bark)	CHCl_3_ (ref. 115)
	*E. mildbraedii* (root bark)	EtOAc^51^
	*E. sigmoidea* (stem bark)	MeOH^99^
	*E. abyssinica* (twigs and roots)	CHCl_3_ : MeOH (1 : 1)^86^
	*E. addisoniae* (stem bark)	CH_2_Cl_2_ (ref. 80)
Abyssinone V (5)	*E. sigmoidea* (stem bark)	CHCl_3_ (ref. 62)
	*E. abyssinica* (root bark)	EtOAc^123^
	*E. eriotricha* (stem bark)	CHCl_3_ (ref. 117)
	*E. abyssinica* (stem bark)	MeOH^89^
	*E. mildbraedii* (root bark)	EtOAc^51^
	*E. abyssinica* (twigs and roots)	CHCl_3_ : MeOH (1 : 1)^86^
	*E. melanacantha* (stem bark)	CH_2_Cl_2_ (ref. 110)
	*E. addisoniae* (stem bark)	CH_2_Cl_2_ (ref. 80)
	*E. burttii* (stem bark)	CHCl_3_ (ref. 159)
Sigmoidin A (6)	*E. abyssinica* (stem bark)	MeOH^89^
	*E. latissima* (stem bark)	CHCl_3_ : MeOH (1 : 1)^83^
	*E. sigmoidea* (stem bark)	EtOAc^68^
	*E. abyssinica* (stem bark)	MeOH^122^
	*E. latissima* (stem bark)	MeOH^84^
Sigmoidin B (7)	*E. sigmoidea* (stem bark)	EtOAc^33^
	*E. abyssinica* (stem bark)	MeOH^58^
	*E. latissima* (stem bark)	MeOH^84^
	*E. latissima* (stem bark)	CHCl_3_ : MeOH (1 : 1)^83^
	*E. abyssinica* (stem bark)	MeOH^122^
	*E. abyssinica* (twigs and roots)	CHCl_3_ : MeOH (1 : 1)^86^
Sigmoidin C (8)	*E. eriotricha* (stem bark)	CHCl_3_ (ref. 138)
	*E. abyssinica* (stem bark)	MeOH^89^
	*E. latissima* (stem bark)	MeOH^84^
	*E. abyssinica* (stem bark)	MeOH^122^
	*E. latissima* (stem bark)	CHCl_3_ : MeOH (1 : 1)^83^
	*E. abyssinica* (twigs and roots)	CHCl_3_ : MeOH (1 : 1)^86^
Sigmoidin D (9)	*E. abyssinica* (stem bark)	MeOH^87^
	*E. latissima* (stem bark)	MeOH^84^
	*E. abyssinica* (twigs and roots)	CHCl_3_ : MeOH (1 : 1)^86^
Sigmoidin E (10)	*E. mildbraedii* (root bark)	EtOAc^51^
	*E. sacleuxii* (stem bark)	EtOAc and CHCl_3_ (ref. 73 and 76)
	*E. abyssinica* (stem bark)	EtOAc^127^
Sigmoidin F (11)	*E. abyssinica* (stem bark)	MeOH^89^
	*E. abyssinica* (stem bark)	MeOH^122^
	*E. latissima* (stem bark)	CHCl_3_ : MeOH (1 : 1)^83^
	*E. abyssinica* (twigs and roots)	CHCl_3_ : MeOH (1 : 1)^86^
	*E. latissima* (stem bark)	MeOH^84^
Sigmoidin G (12)	*E. sigmoidea* (stem bark)	EtOAc^68^
Sigmoidin L (14)	*E. sigmoidea* (stem bark)	EtOAc^68^
Abyssinone V 4′-*O*-methyl ether (18)	*E. caffra* (stem bark)	Acetone^8^
		EtOAc^118^
	*E. addisoniae* (stem bark)	CH_2_Cl_2_ (ref. 80)
	*E. schliebenii* (stem bark)	MeOH^112^
	*E. mildbraedii* (root bark)	EtOAc^51^
	*E. lysistemon* (stem bark)	CH_2_Cl_2_ (ref. 71)
	*E. sacleuxii* (stem bark)	EtOAc and CHCl_3_ (ref. 73 and 76)
	*E. burttii* (stem bark)	CHCl_3_ (ref. 143 and 160)
	*E. burttii* (stem bark)	Acetone^124^
	*E. droogmansiana* (root bark)	EtOAc^96^
	*E. melanacantha* (stem bark)	CH_2_Cl_2_ (ref. 110)
Senegalensein (23)	*E. senegalensis* (stem bark)	CHCl_3_ (ref. 103)
	*E. caffra* (stem bark)	EtOAc^130^
	*E. indica* (stem bark)	CH_2_Cl_2_ : MeOH (1 : 1)^69^
	*E. lysistemon* (stem bark)	CH_2_Cl_2_ (ref. 71)
	*E. addisoniae* (stem bark)	MeOH^77^
Abyssinin III (27)	*E. abyssinica* (stem bark)	MeOH^91^
	*E. latissima* (stem bark)	MeOH^84^
Burttinone (28)	*E. caffra* (stem bark)	EtOAc^130^
	*E. lysistemon* (stem bark)	CH_2_Cl_2_ (ref. 71)
	*E. sacleuxii* (stem bark)	EtOAc and CHCl_3_ (ref. 73 and 76)
	*E. burttii* (stem bark)	CHCl_3_ (ref. 143 and 160)
	*E. burttii* (stem bark)	Acetone^124^
	*E. schliebenii* (stem bark)	MeOH^112^
Erycaffra D (29)	*E. caffra* (stem bark)	EtOAc^130^
Erycaffra F (30)	*E. caffra* (stem bark)	EtOAc^130^
Erylatissin C (40)	*E. abyssinica* (stem bark)	MeOH^91^
	*E. abyssinica* (twigs and roots)	CHCl_3_ : MeOH (1 : 1)^86^
Abyssinoflavanone VII (43)	*E. addisoniae* (stem bark)	CH_2_Cl_2_ (ref. 80)
5-Deoxyabyssinin II (44)	*E. abyssinica* (root bark)	EtOAc^92^
	*E. abyssinica* (stem bark)	EtOAc^127^
Sigmoidin B-4′-methyl ether (68)	*E. livingstoniana* (stem bark)	EtOAc^93^
	*E. burttii* (stem bark)	CHCl_3_ (ref. 143 and 160)
	*E. melanacantha* (stem bark)	CH_2_Cl_2_ (ref. 110)
	*E. burttii* (stem bark)	CHCl_3_ (ref. 159)
Carpachromene (95)	*E. vogelii* (stem bark)	CH_2_Cl_2_ : MeOH (1 : 1)^126^
	*E. senegalensis* (stem bark)	CH_2_Cl_2_ (ref. 107)
Limonianin (98)	*E. vogelii* (stem bark)	CH_2_Cl_2_ : MeOH (1 : 1)^126^
Abyssinone VI (100)	*E. abyssinica* (stem bark)	MeOH^122^
Isobavachalcone (103)	*E. burttii* (stem bark)	CHCl_3_ (ref. 143 and 160)
Licoagrochalcone A (104)	*E. abyssinica* (stem bark)	EtOAc^127^
	*E. abyssinica* (stem bark)	MeOH^122^
Eriotrichin B/bidwillon A (110)	*E. eriotricha* (stem bark)	MeOH^129^
	*E. burttii* (stem bark)	CHCl_3_ (ref. 160)
	*E. sigmoidea* (stem bark)	MeOH^99^
	*E. lysistemon* (root bark)	CH_2_Cl_2_ (ref. 142)
	*E. burttii* (stem bark)	CHCl_3_ (ref. 159)
2,3-Dihydroauriculatin (117)	*E. addisoniae* (stem bark)	MeOH^77^
Sigmoidin I (118)	*E. sigmoidea* (stem bark)	MeOH^99^
Lysisteisoflavanone (113)	*E. lysistemon* (stem bark)	CH_2_Cl_2_ (ref. 71)
Orientanol E (122)	*E. lysistemon* (root bark)	CH_2_Cl_2_ (ref. 142)
Sophoraisoflavanone A (133)	*E. droogmansiana* (root bark)	CH_2_Cl_2_ : MeOH (1 : 1)^97^
8-Prenylluteone (139)	*E. senegalensis* (stem bark)	MeOH^104^
	*E. senegalensis* (stem bark)	CH_2_Cl_2_ (ref. 100 and 101)
	*E. vogelii* (stem bark)	CH_2_Cl_2_ : MeOH (1 : 1)^126^
Warangalone/scandenone (141)	*E. senegalensis* (stem bark)	MeOH^140^
	*E. vogelii* (stem bark)	CH_2_Cl_2_ : MeOH (1 : 1)^126^
	*E. caffra* (stem bark)	EtOAc^130^
	*E. mildbraedii* (root bark)	EtOAc^55^
	*E. mildbraedii* (stem bark)	CH_2_Cl_2_ (ref. 53)
	*E. vogelii* (leaves)	EtOH^57^
	*E. sigmoidea* (stem bark)	MeOH^65^
	*E. sigmoidea* (stem bark)	EtOAc^68^
	*E. addisoniae* (stem bark)	MeOH^77^
	*E. senegalensis* (stem bark)	CHCl_3_ (ref. 105)
Alpinumisoflavone (148)	*E. senegalensis* (stem bark)	CH_2_Cl_2_ (ref. 100 and 101)
	*E. caffra* (stem bark)	EtOAc^130^
	*E. indica* (stem bark)	CH_2_Cl_2_ : MeOH (1 : 1)^69^
	*E. lysistemon* (stem bark)	CH_2_Cl_2_ (ref. 71)
	*E. lysistemon* (twigs)	EtOAc^72^
6,8-Diprenylgenistein (149)	*E. senegalensis* (stem bark)	MeOH^104^
	*E. senegalensis* (stem bark)	CH_2_Cl_2_ (ref. 100 and 101)
	*E. mildbraedii* (root bark)	EtOAc^51^
	*E. vogelii* (stem bark)	CH_2_Cl_2_ : MeOH (1 : 1)^126^
	*E. sigmoidea* (stem bark)	MeOH^65^
	*E. sigmoidea* (stem bark)	EtOAc^68^
	*E. lysistemon* (twigs)	EtOAc^72^
	*E. senegalensis* (stem bark)	CHCl_3_ (ref. 98)
	*E. excels* (stem bark)	EtOH : H_2_O (8 : 2)^9^
	*E. senegalensis* (stem bark)	EtOH : H_2_O (3 : 2)^106^
Erysenegalensein E (151)	*E. caffra* (stem bark)	EtOAc^130^
	*E. indica* (stem bark)	CH_2_Cl_2_ : MeOH (1 : 1)^69^
	*E. indica* (stem bark)	CH_2_Cl_2_ : MeOH (1 : 1)^69^
	*E. lysistemon* (twigs)	EtOAc^72^
	*E. lysistemon* (stem bark)	CH_2_Cl_2_ (ref. 71)
Auriculatin (152)	*E. senegalensis* (stem bark)	MeOH^140^
	*E. caffra* (stem bark)	EtOAc^49^
	*E. vogelii* (stem bark)	CH_2_Cl_2_ : MeOH (1 : 1)^126^
	*E. senegalensis* (stem bark)	CHCl_3_ (ref. 98)
Derrone (155)	*E. caffra* (stem bark)	EtOAc^130^
	*E. lysistemon* (twigs)	EtOAc^72^
Erysenegalensein M (159)	*E. mildbraedii* (stem bark)	CH_2_Cl_2_ (ref. 53)
Erycaffra C (160)	*E. caffra* (stem bark)	EtOAc^130^
Isoerysenegalensein E (161)	*E. caffra* (stem bark)	EtOAc^130^
	*E. lysistemon* (stem bark)	CH_2_Cl_2_ (ref. 71)
	*E. lysistemon* (twigs)	EtOAc^72^
Isosenegalensein (162)	*E. caffra* (stem bark)	EtOAc^130^
	*E. lysistemon* (stem bark)	CH_2_Cl_2_ (ref. 71)
Erythrinin C (163)	*E. caffra* (stem bark)	EtOAc^130^
Erysubin B (164)	*E. caffra* (stem bark)	EtOAc^130^
Parvisoflavone B (172)	*E. schliebenii* (root bark)	MeOH^112^
Euchrenone b10 (174)	*E. addisoniae* (root bark)	MeOH^78^
Vogelin C (176)	*E. droogmansiana* (root bark)	CH_2_Cl_2_ : MeOH (1 : 1)^97^
Isolupalbigenin (181)	*E. droogmansiana* (root bark)	CH_2_Cl_2_ : MeOH (1 : 1)^97^
Corylin (190)	*E. sacleuxii* (root bark)	Acetone^75^
Neobavaisoflavone (189)	*E. sigmoidea* (stem bark)	MeOH^99^
	*E. latissimi* (stem wood)	CHCl_3_ : MeOH (1 : 1)^82^
	*E. abyssinica* (twigs and roots)	CHCl_3_ : MeOH (1 : 1)^86^
	*E. senegalensis* (root bark)	CH_2_Cl_2_ : MeOH (1 : 1)^108^
Dimethylalpinumisoflavone (194)	*E. indica* (stem bark)	CH_2_Cl_2_ : MeOH (1 : 1)^69^
Wighteone (197)	*E. lysistemon* (stem bark)	CH_2_Cl_2_ (ref. 71)
Erysubin F (203)	*E. addisoniae* (root bark)	MeOH^78^
	*E. brucei* (root bark)	CH_2_Cl_2_–MeOH (1 : 1)^94^
5′-Prenylpratensein (200)	*E. latissimi* (stem bark)	CHCl_3_ : MeOH (1 : 1)^83^
	*E. abyssinica* (twigs and roots)	CHCl_3_ : MeOH (1 : 1)^86^
	*E. burttii* (stem bark)	Acetone^124^
	*E. schliebenii* (root bark)	CH_2_Cl_2_ (ref. 112)
3′-Prenylbiochanin A (201)	*E. schliebenii* (stem bark)	MeOH^112^
Erythrabyssin I/cristacarpin (234)	*E. droogmansiana* (root bark)	EtOAc^125^
	*E. lysistemon* (stem bark)	MeOH^70^
	*E. lysistemon* (leaves)	CHCl_3_ (ref. 72)
	*E. latissimi* (stem wood)	CHCl_3_ : MeOH (1 : 1)^82^
	*E. brucei* (root bark)	CH_2_Cl_2_–MeOH (1 : 1)^94^
	*E. droogmansiana* (root bark)	CH_2_Cl_2_ : MeOH (1 : 1)^97^
	*E. abyssinica* (stem bark)	EtOAc^146^
	*E. burana* (stem bark)	CHCl_3_ (ref. 85)
Erythrabyssin II (235)	*E. mildbraedii* (root bark)	EtOH^56^
	*E. sigmoidea* (root bark)	MeOH^132^
	*E. abyssinica* (twigs, roots)	CHCl_3_ : MeOH (1 : 1)^86^
	*E. abyssinica* (stem bark)	EtOAc^91^
	*E. brucei* (root bark)	CH_2_Cl_2_–MeOH (1 : 1)^94^
	*E. burttii* (root bark)	Acetone^143^
	*E. lysistemon* (root bark)	CH_2_Cl_2_ (ref. 142)
Phaseollin (236)	*E. lysistemon* (stem bark)	MeOH^70^
	*E. burttii* (root bark)	Acetone^143^
	*E. senegalensis* (stem bark)	CH_2_Cl_2_ (ref. 107)
	*E. melanacantha* (stem bark)	CH_2_Cl_2_ (ref. 111)
	*E. abyssinica* (stem bark)	EtOAc^146^
	*E. lysistemon* (root bark)	CH_2_Cl_2_ (ref. 142)
Phaseollidin (237)	*E. sigmoidea* (root bark)	MeOH^129^
	*E. burana* (bark)	CHCl_3_ (ref. 85)
	*E. lysistemon* (leaves)	CHCl_3_ (ref. 72)
	*E. latissimi* (stem wood)	CHCl_3_ : MeOH (1 : 1)^82^
	*E. abyssinica* (twigs, roots)	CHCl_3_ : MeOH (1 : 1)^86^
	*E. abyssinica* (stem bark)	EtOAc^145^
	*E. burttii* (root bark)	Acetone^143^
	*E. droogmansiana* (root bark)	EtOAc^125^
	*E. droogmansiana* (root bark)	CH_2_Cl_2_ : MeOH (1 : 1)^97^
	*E. melanacantha* (stem bark)	CH_2_Cl_2_ (ref. 111)
	*E. lysistemon* (root bark)	CH_2_Cl_2_ (ref. 142)
Erybraedin A (238)	*E. eriotricha* (stem bark)	MeOH^129^
	*E. eriotricha* (root bark)	CH_2_Cl_2_ (ref. 129)
	*E. lysistemon* (stem bark)	MeOH^70^
	*E. burttii* (root bark)	Acetone^143^
	*E. senegalensis* (stem bark)	CH_2_Cl_2_ (ref. 107)
	*E. melanacantha* (stem bark)	CH_2_Cl_2_ (ref. 111)
	*E. lysistemon* (root bark)	CH_2_Cl_2_ (ref. 142)
Erybraedin B (239)	*E. abyssinica* (stem bark)	EtOAc^145^
	*E. lysistemon* (root bark)	CH_2_Cl_2_ (ref. 142)
Erybraedin C (240)	*E. eriotricha* (stem bark)	MeOH^129^
	*E. eriotricha* (root bark)	CH_2_Cl_2_ (ref. 144)
	*E. abyssinica* (stem bark)	EtOAc^145^
	*E. senegalensis* (stem bark)	CH_2_Cl_2_ (ref. 107)
Isoneorautenol (241)	*E. eriotricha* (stem bark)	MeOH^129^
	*E. eriotricha* (root bark)	CH_2_Cl_2_ (ref. 144)
	*E. lysistemon* (stem bark)	MeOH^70^
	*E. livingstoniana* (root bark)	CH_2_Cl_2_ : MeOH (1 : 1)^141^
	*E. excelsa* (root bark)	CH_2_Cl_2_ : MeOH (1 : 1)^108^
	*E. melanacantha* (stem bark)	CH_2_Cl_2_ (ref. 111)
	*E. abyssinica* (stem bark)	EtOAc^146^
Erybraedin D (242)	*E. eriotricha* (root bark)	CH_2_Cl_2_ (ref. 144)
	*E. abyssinica* (stem bark)	EtOAc^145^
	*E. senegalensis* (stem bark)	CH_2_Cl_2_ (ref. 107)
Erybraedin E (243)	*E. eriotricha* (root bark)	CH_2_Cl_2_ (ref. 144)
Neorautenol (244)	*E. abyssinica* (stem bark)	EtOAc^91^
	*E. burttii* (stem bark)	CHCl_3_ (ref. 160)
	*E. schliebenii* (root bark)	CH_2_Cl_2_ (ref. 112)
	*E. abyssinica* (stem bark)	EtOAc^146^
	*E. burttii* (stem bark)	CHCl_3_ (ref. 159)
Sandwicensin (245)	*E. brucei* (root bark)	CH_2_Cl_2_–MeOH (1 : 1)^94^
Calopocarpin (249)	*E. lysistemon* (stem bark)	MeOH^70^
	*E. livingstoniana* (root bark)	CH_2_Cl_2_ : MeOH (1 : 1)^141^
	*E. brucei* (root bark)	CH_2_Cl_2_ : MeOH (1 : 1)^94^
	*E. burttii* (stem bark)	CHCl_3_ (ref. 160)
	*E. burttii* (root bark)	Acetone^143^
	*E. abyssinica* (stem bark)	EtOAc^146^
	*E. burttii* (stem bark)	CHCl_3_ (ref. 159)
Erysubin D (254)	*E. abyssinica* (stem bark)	EtOAc^146^
Eryvarin D (255)	*E. abyssinica* (stem bark)	EtOAc^146^
	*E. lysistemon* (root bark)	CH_2_Cl_2_ (ref. 142)
	*E. abyssinica* (root bark)	Acetone^127^
Shinpterocarpin (257)	*E. senegalensis* (stem bark)	CH_2_Cl_2_ (ref. 107)
	*E. sacleuxii* (root bark)	Acetone^75^
	*E. abyssinica* (root bark)	Acetone^161^
Sophorapterocarpan A (258)	*E. abyssinica* (stem bark)	EtOAc^145^
	*E. abyssinica* (stem bark)	EtOAc^146^
	*E. melanacantha* (stem bark)	CH_2_Cl_2_ (ref. 111)
Eryvarin K (262)	*E. senegalensis* (stem bark)	CH_2_Cl_2_ (ref. 107)
	*E. lysistemon* (root bark)	CH_2_Cl_2_ (ref. 142)
Erysubin E (265)	*E. brucei* (root bark)	CH_2_Cl_2_–MeOH (1 : 1)^94^
	*E. abyssinica* (stem bark)	EtOAc^146^
Erystagallin A (266)	*E. droogmansiana* (root bark)	CH_2_Cl_2_ : MeOH (1 : 1)^97^
	*E. abyssinica* (stem bark)	EtOAc^146^
Erycristagallin (269)	*E. mildbraedii* (root bark)	EtOAc^55^
	*E. abyssinica* (root bark)	Acetone^127^
	*E. abyssinica* (root bark)	Acetone^161^
	*E. burttii* (stem bark)	CHCl_3_ (ref. 159)

## 5 Characterization of prenyl moieties

Several spectroscopy methods are commonly used in the characterization of flavonoids, such as Circular Dichroism, Infrared and NMR spectroscopy.^162^ In general, NMR spectroscopy is helpful in the identification of prenyl moieties; this is fundamentally because the characteristics of prenyl groups in UV and IR spectroscopy interfere with those of the flavonoids. The first noticeable characteristics of prenyl groups in ^1^H NMR spectra are the chemical shifts of protons of the hydroxyl groups; these signals resonate between 5 and 8 ppm. In 2-hydroxy-3-methylbut-3-enyl the configuration of the double bond is usually *trans*, as evidenced by coupling constants *J* = 16 Hz.^93^ In chromans, furanose or any open prenyl groups containing asymmetric carbon, the wide ranges in chemical shifts could be due to the absolute configurations, but unfortunately these configurations were not all determined. Meanwhile the configuration of C-3′′ in sigmoidin D (9) was determined by using the Horeaus' method with (+) 2-phenylbutanoic anhydride, and was attributed to “*S*”.^61^ In addition, the positions of these prenyl moieties relative to aromatic rings can equally affect the chemical shift values. Tables 3 and 4 provide the chemical shift ranges characterizing some commonly occurring prenyl groups.

**Table 3:** Chemical shift ranges characteristic of open prenyl groups

Prenyl groups	Position, *δ*_C_ and *δ*_H_ in ppm					References
	1	2	3	4	5	
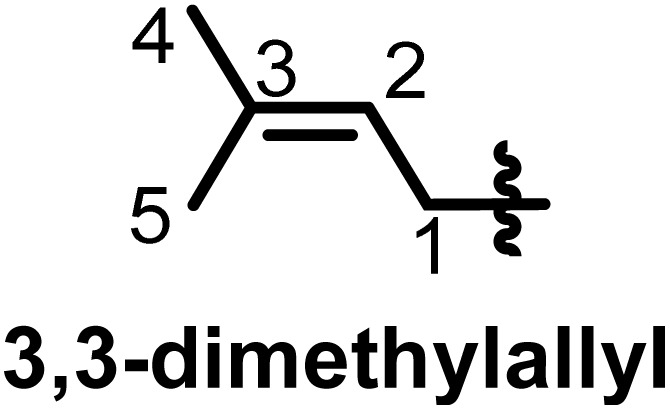	21–22	121–122	132–136	25–26	17–18	57, 119, 67, 82, 77 and 51
	3.15–3.45	5.00–5.3		1.50–1.80	1.50–1.80	
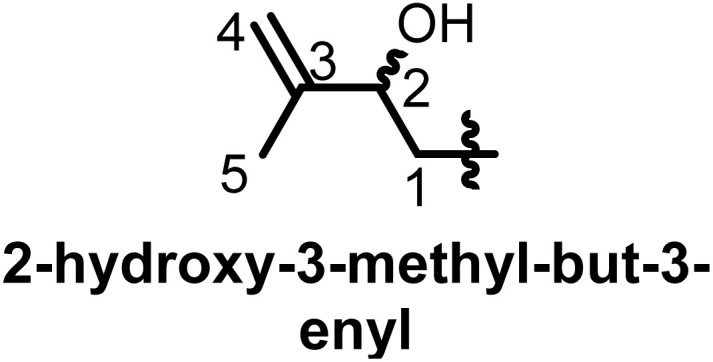	28–34	74–78	145–150	109–112	17–19	69, 80, 100, 112 and 163
	2.80–3.30	4.00–4.50		4.70–5.00	1.75–1.85	
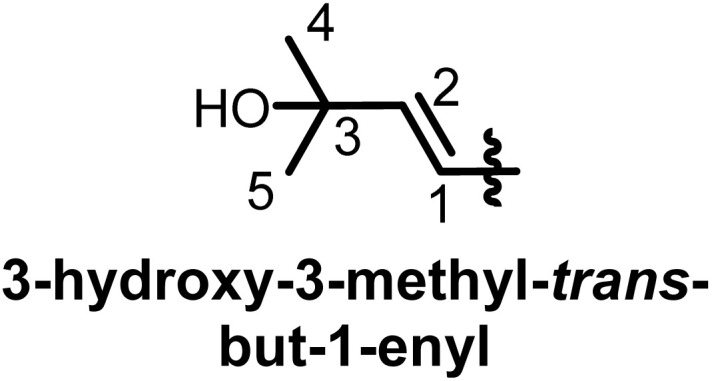	120–125	135–142	70–83	25–31	25–31	51, 141 and 151
	6.80–6.95	6.30–6.60		1.30–1.50	1.30–1.50	
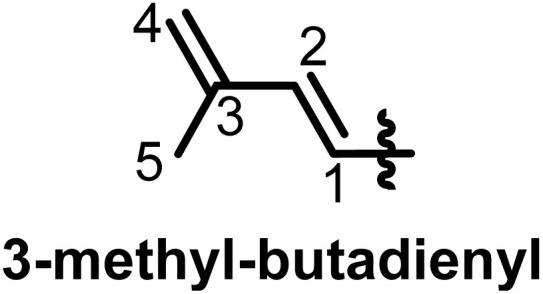	122–124	133–135	142–44	118–119	18–19	112 and 151
	6.70–6.90	6.40–7.00		5.00–5.2	1.8–2.00	
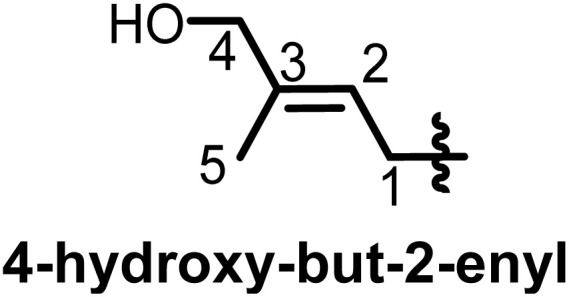	32	129.5	140	72.4	18.5	131
	3.4	5.4		3.95	1.80	
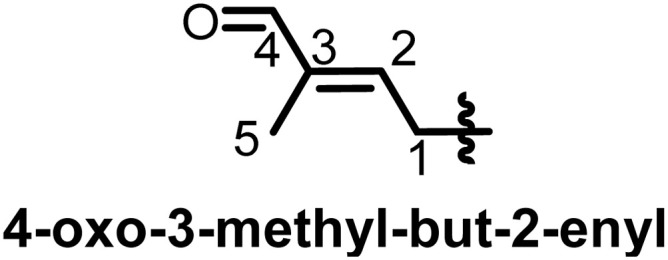	31	154	141	198	10.5	53
	3.6	6.5	9.3		1.8	
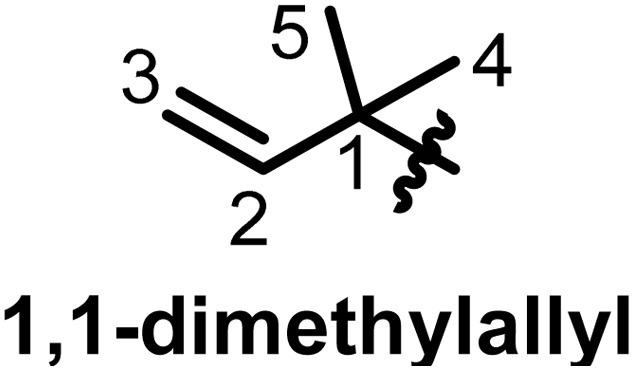	39	148	113.3	27.1	27.1	143
		6.2	5.33	1.4	1.4	

**Table 4:** Chemical shift ranges characteristic of heterocyclic prenyl groups

Prenyl groups	Position, *δ*_C_ and *δ*_H_ in ppm					References
	2	3	4	5	6	
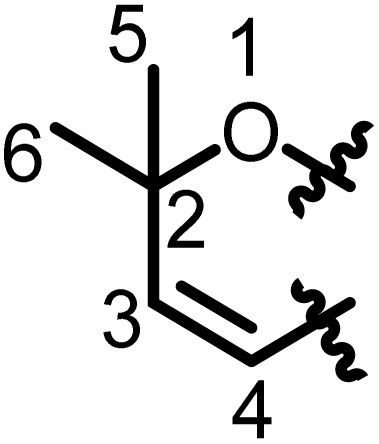	76–80	127–132	115–123	27–29	27–29	52, 67, 82, 90, 97 and 98
		5.40–5.60	6.20–6.65	1.40–1.50	1.40–1.50	
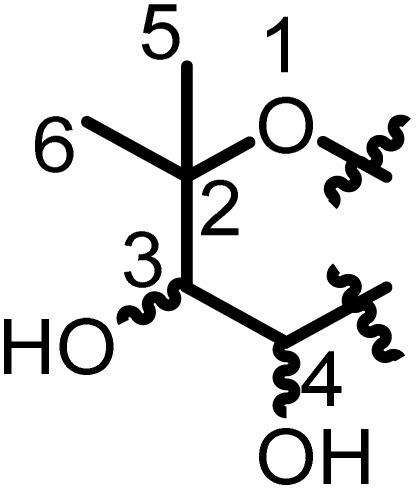	79–81	76–77	68–70	25–28	19–20	66, 90 and 128
		3.50–3.60	4.50–4.60	1.20–1.25	1.40–1.55	
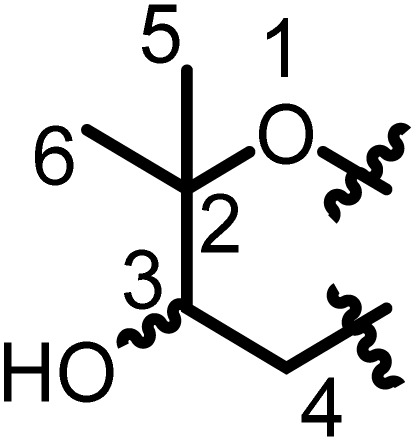	76–80	69–70	26–33	25–27	20–21	87, 90, 92, 128, 131 and 146
		3.65–3.85	2.40–2.80	1.30–1.40	1.10–1.27	
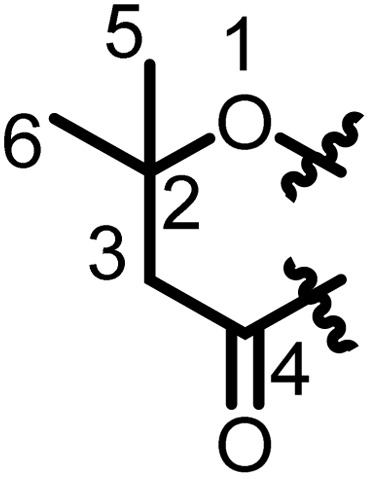	81.1	49.3	192.2	25.6	25.6	90
		2.80		1.48	1.48	
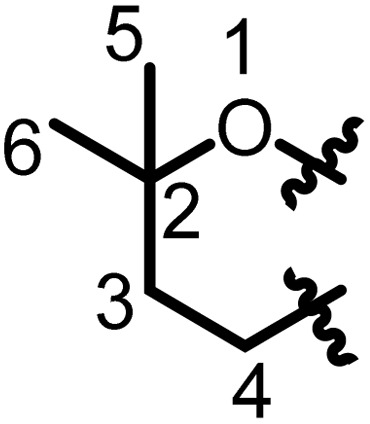	75–76	32–34	23–24	26–27	26–28	54 and 87
		1,80–1.90	2.75–2.85	1.30–140	1.30–140	
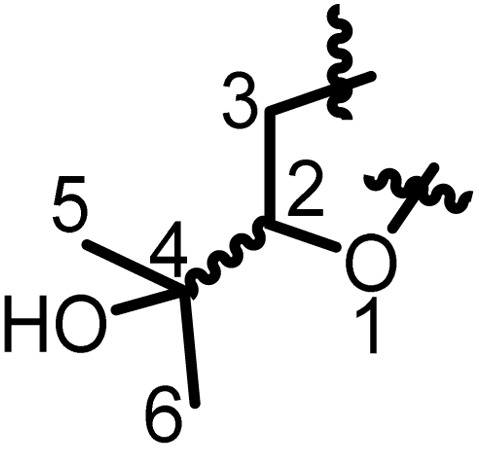	90–92	26–32	71–73	25–27	24–26	57, 91, 126, 138 and 142
	4.60–3.75	3.00–3.25		1.25–1.30	1.20–1.27	
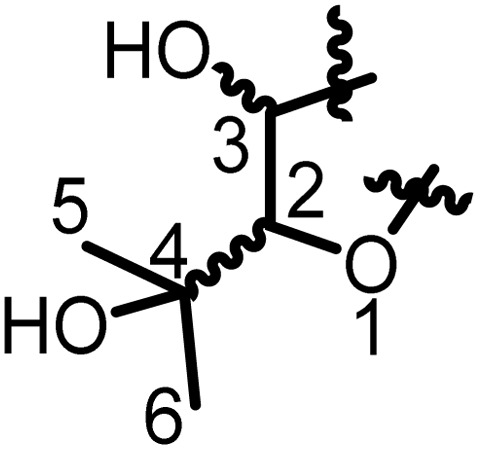	97–98	73–75	71–72	25–27	25–27	91
	3.30–3.50	5.10–5.40		1.65–1.72	1.65–1.72	

## 6 Biological activities

Flavonoids represent a major class of natural products from plants, and given that many plant-derived flavonoids are commonly used as food, they are generally considered to be non-toxic to human organisms.^164^ Therefore, targeting their importance as bioactive ingredients should be beneficial to humans, both as drugs and for food supplements. Recent reports have stated that the substitution of the flavonoid ring system by a prenyl side chain confers a strong affinity to biological membranes to the molecule due to the increase of their lipophilicity.^165^

### 6.1 Antimicrobial activity

Some reported data have suggested that flavonoids mainly interact with the cell membranes of Gram-positive bacteria. These suggestions were supported by the study of Juang *et al.*; the lipophilicity might be a key factor which increases the activity against Gram positive bacteria.^6^ However, it is reported that some modifications such as carbonylation, hydroxylation and methoxylation and/or cyclisation of the prenyl and/or geranyl side chains negatively impact the activity.^166^

Abyssinone V 4′-*O*-methyl ether (18), 6,8-diprenylgenistein (149), alpinumisoflavone (148) and burttinone (28) were assayed for their effect on bacteria growth using the microdilution method. Results showed that 18 and 148 had significative antibacterial activity on *E. coli* ATCC 11775 and *K. pneumonia*, with the same MIC value of 3.9 μg mL^−1^ (9.24 and 11.6 μM, respectively) compared to the positive control neomycin (MIC = 1.6 μg mL^−1^, 2.6 μM). Alpinumisoflavone (148) equally showed the same activity against *S. aureus* (neomycin MIC = 0.8 μg mL^−1^, 1.3 μM) whereas 6,8-diprenylgenistein (149) exhibited an activity of 7.8 μg mL^−1^ (19.2 μM) towards the growth of *S. aureus* ATCC 12600, *E. coli*, *K. pneumonia* (ATCC 13883) and 15.6 against *B. subtilis* ATCC 6051. The positive control was in the range 0.78 to 1.6 μg mL^−1^. *B. subtilis* was mainly affected by compound 149, only with a MIC of 7.8 μg mL^−1^ (19.2 μM) relative to the MIC value of 1.3 μM exhibited by neomycin. Given that compound 28 was the less active (31 < MIC < 125 μg mL^−1^), the hydroxylation of the prenyl group at 3′ could have been more negatively affected than 148 which has no hydroxyl on its prenyl groups.^8^ Sandwicensin (245) and 3*R* (−) erythbidin A (223) when evaluated for their effect on *S. aureus* ATCC6538P both showed a MIC value of 62.5 μg mL^−1^ (183.8 μM) (positive control not indicated).^50^ Abyssinone VII (71), abyssinone IV (4), abyssinone V (5), phaseollidin (237), erythrabyssin II (236), sigmoidin B (7), sigmoidin C (8), sigmoidin D (9), sigmoidin F (11), 5′-prenylpratensein (200), sandwicensin (245), neobavaisoflavone (189), semilicoisoflavone B (212) and licoagrochalcone A (104) were tested against some microbes using the bioautographic agar overlay method, and all these compounds strongly inhibited the growth of *B. subtilis* and *S. aureus* with MIC values in the range of 0.5 to 4 μg mL^−1^ relative to chloramphenicol (MIC = 0.001 μg mL^−1^). Only sigmoidin B (7) and Abyssinone VII (71) strongly affected the growth of *E. coli*, with a MIC value of 5.0 μg mL^−1^ (14.0 and 14.7 μM) (MIC = 0.05 μg mL^−1^ or 0.05 μM for chloramphenicol). Sigmoidin C (8), abyssinone VII (71), erythrabyssin II (235), phaseollidin (237), sandwicensin (245), neobavaisoflavone (189), 5′-prenylpratensein (200) and semilicoisoflavone B (212) exhibited strong activities against *Saccharomyces cerevisiae* with MIC values of 0.5, 5.0, 2.0, 0.5, 1.0, 0.5, 0.5, and 3.0 μg mL^−1^, respectively (MIC = 0.005 μg mL^−1^ for miconazole).^86^ It has been also reported that sigmoidin L (14) inhibited the growth of *S. aureus* and *P. vulgaris* with respective MICs of 4.0 and 7.0 μg mL^−1^ (11.3 and 17.8 μM), however, no positive control was reported in this study.^116^ Additionally, its congener sigmoidin M (16) exhibited significant antibacterial activity against *S. aureus* with a MIC of 4.0 μg mL^−1^ or 10.3 μM (no positive control).^66^ Erybraedins A–C (238–240), erythrabyssin II (235) and isoneorautenol (241) were assayed for their effect on the growth of certain bacteria, and erythrabyssin II (235) potentially inhibited the growth of *S aureus* ATCC 13709 and *Mycobacterium smegmatis* ATCC 607, with MIC values of 3.12 and 0.78 μg mL^−1^ (8.8 and 2.2 μM), respectively, in comparison to tests against streptomycin with MICs of 5.0 and 1.25 μg mL^−1^ (8.6 and 2.1 μM), respectively. Other compounds showed MICs in the range 6.25 and 25 μg mL^−1^ against these two bacteria and none of these compounds affected the growth of, *E. coli* ATCC 9637, *Salmonella gallinarum* ATCC 9184, *Klebsiella pneumoniae* ATCC 10031, *Candida albicans* ATCC 10231 or *Pseudomonas aeruginosa* ATCC 27853.^56^ Erybraedin A (238), erythrabyssin II (235), erystagallin A (266) and erycristagallin (269) exhibited strong antibacterial activities against several species and strains of *Streptococcus* and *Staphylococcus* as well as several strains of MRSA (Multi Resistant *Staphylococcus aureus*), with MICs ranging from 0.78 to 6.25 μg mL^−1^, relative to vancomycin and oxacillin (0.09 < MIC < 256 μg mL^−1^). These are promising results with regard to the fight against antibacterial resistance, and these compounds represent some potential antibiotics isolated from medicinal plants.^167^ Brucein B (130) displayed moderate antibacterial activities, with MIC of 62 μg mL^−1^ or 167.6 μM against *Bacillus cereus* (ATCC 33019) compared to chloramphenicol (MIC = 15 μg mL^−1^, 46.4 μM).^94^ The five compounds isolated from *E. livingstoniana* were evaluated for their antibacterial activity. Sigmoidin B-4′-methyl ether (68) displayed a good activity with MIC of 5.0 μg mL^−1^ against *E. coli* (DSM 1116), *B. subtilis* (DSM 1088), and *E. coli* (DSM 682). 7,3 ′-dihydroxy-4′-methoxy-5′-prenylflavanone (78) showed a MIC value of 2.0 μg mL^−1^ or 5.4 μM against *B. subtilis* (DSM 1088) in comparison to Streptomycin and gentamycin with MIC = 5.0 μg mL^−1^ or 8.6 μM and 1.0 μg mL^−1^ or 2.1 μM and a MIC of 5 μg mL^−1^ or 13.5 μM against the two strains of *E. coli* (streptomycin and gentamycin MIC = 1.0 μg mL^−1^ or 1.7 and 2.1 μM).^93^ Erythrabyssins I (234) and II (235), phaseollin (236), phaseollidin (237), abyssinone I (1), abyssinone II (2), abyssinone III (3), abyssinone IV (4), abyssinone V (5) and abyssinone VI (100) were assessed for their antimicrobial activities. Erythrabyssin I (234) and phaseollin (236) showed MICs of 12.5 and 6.25 μg mL^−1^, respectively, against *S. aureus* and *B. subtilis*. Erythrabyssin II (235) exhibited strong antibacterial activity against *S. aureus*, *B. subtilis* and *Micrococcus lysodeikticus* with a MIC value of 3.13 μg mL^−1^, whereas abyssinone V (5) showed a MIC of 12.5 μg mL^−1^ against *Micrococcus lysodeikticus* (positive control was not reported). Regarding antifungal activity, erythrabyssin I (234) showed a MIC of 6.25 μg mL^−1^ or 18.5 μM against *Sclerotinia libertiana* and phaseollin (236) displayed an activity of 12.5 μg mL^−1^ or 31.9 μM against *Sclerotinia libertiana*, *Mucor mucedo* and *Rhizopus chinensis*. In addition, abyssinones I (1) and II (2) exhibited a MIC of 12.5 μg mL^−1^ (32.1 and 38.6 μM) against *Sclerotinia libertiana*.^113^ Sigmoidin I (118), corylin (188), neobavaisoflavone (189) and phaseollidin (237) were assessed for their capacity to affect the growth of *Candida albicans, Cryptococcus neoformans*, *Aspergillus fumigatus* and *S. aureus*. Only neobavaisoflavone (189) showed a MIC of 50 μg mL^−1^ or 156.3 μM against *C. neoformans* and *A. fumigatus*,^129^ and neobavaisoflavone (189) displayed a MIC of 3.2 μg mL^−1^ or 10.0 μM against *S. aureus*.^132^ Sigmoidin A (6) inhibited the growth of *S. aureus*, *M. luteus* (WS) and *M. luteus* (IPC) with MICs of 12.5, 25 and 50 μg mL^−1^ (29.5, 59.0 and 117.9 μM); with inhibition diameter varying from 10 to 13 mm; sigmoidin B (7) only affected the growth of *S. aureus* with the same MICs.^114^ Eriotrichin B (110), erybraedins A (238) and C (240) exhibited good antibacterial activities against *S. aureus* with MICs of 8.3, 13.6, and 12.8 μg mL^−1^ (20.3, 42.0 and 32.8 μM), respectively, relative to a MIC = 6 μg mL^−1^ or 18 μM for penicillin.^144^ Abyssinone IV-4′-methylether (31) exhibited an activity of 25 μg mL^−1^ or 63.8 μM against *S. aureus*, *P. stuartii* ATCC 29916 and *E. aerogenes* ATCC1 3048 relative to 8.0, 128 and 32 μg mL^−1^, respectively, for ciproflaxacin.^125^ Neobavaisoflavone (189) exhibited good to moderate antibacterial activity against *E coli* ATCC8739 (MIC = 8.0 μg mL^−1^ or 25.0 μM), *E. coli* AG100 ΔacrAB mutant AG100, with an over-expressing acrF gene (MIC = 32.0 μg mL^−1^ or 100 μM), *Enterobacter cloacae* Clinical MDR isolates (MIC = 8.0 μg mL^−1^ or 25.0 μM), *Klebsiella pneumonia* Clinical MDR isolate (MIC = 8 μg mL^−1^ or 25.0 μM), *Providencia stuartii* Clinical MDR isolate (MIC = 8 μg mL^−1^ or 25.0 μM) and *Pseudemonas aeruginosa* (MIC = 8.0 μg mL^−1^ or 25.0 μM).^168^ 4′,5,7-Trihydroxy-6-(2′′-hydroxy-3′′-prenyl)isoflavone (207) exhibited a good antimicrobial activity against *E. coli*, *S. aureus* and *Candida mycoderma* with respective MICs of 10.0, 5.0, and 10.0 μg mL^−1^ (29.8, 14.9 and 29.8 μM). 6,8-diprenylgenistein (149) inhibited the growth of *S. aureus* and *C*. *mycoderma* at respective MICs of 1.0 and 5.0 μg mL^−1^ (2.4 and 12.3 μM), erysenegalensein E (151) and isoerysenegalensein E (161) inhibited the growth of *E. coli*, *B. subtilis* and *C. mycoderma* at MIC values of 10.0, 5.0, and 10.0 μg mL^−1^, respectively. Sandwicensin (245) showed a MIC of 10.0 μg mL^−1^ or 31.0 μM against *E. coli* comparatively to chloramphenicol and miconazole had respective MICs of 0.01 and 1.0 μg mL^−1^ against these microorganisms.^72^ Erysubin E (265) showed an IC_50_ of 1.30 μM against *C. perfringens*, cristacarpin (234), and erystagallin A (266) exhibited a good antibacterial activity with IC_50_ values of 2.28 and 2.04 μM against *C. perfringens*. In addition, eryvarin D (255) and erythribyssin O (275) exhibited antibacterial activity with IC_50_s of 2.09 and 1.32 μM, as well as 3.30 and 0.35 μM, respectively, against *C. perfringens* and *V. cholera* comparative to quercetin (IC_50_ = 25.34 μM).^146^ The poor activity of phaseollin (236) could result from the cyclisation of the prenyl group relative to 255 and 275.

### 6.2 Antioxidant activity

Mildbone (33) and mildbenone (102) demonstrated strong scavenging activity with respective IC_50_ values of 20.2 and 28.5 μM compared to the positive control butylated hydroxyanisole (IC_50_ = 44.2 μM).^54^ Sigmoidin A (6) and B (7) showed DPPH scavenging activity at a dose of 100 μM. This activity was found to be better than that of the similar flavonoid (quercetin 3-*O*-β-d-glucopyranoside) with the same hydroxyl pattern, illustrating that the prenyl group might contribute to their scavenging effect.^64^ In addition, these two compounds were assessed for their effect in reducing superoxide anion radicals produced by rat alveolar macrophages *in vivo*, and they inhibited the superoxide anion at 90% and 65% respectively for sigmoidins A (6) and B (7) at a concentration of 100 μM each. The high effect of sigmoidin A (6) might be associated with the contribution of its second prenyl group at 6'.^169^ The isolated compounds from *E. brucei*, namely, bruceins A (129) and B (130), kenusanone F (131), sophoraisoflavanone A (133), cristacarpin (234), eryvarin J (267) and erycristagallin (269) were equally evaluated for their scavenging capacity, and they showed activities with IC_50_ values of 6.3, 13.3, 22.2, 21.2, 6.4, 1.4, and 1.1 μM relative to trolox (IC_50_ = 0.62 μM).^94^ Similarly, erylivingstone B (75), erylivingstone C (76), 5,7-dihydroxy-3′,4′-dimethoxy-5′-(3-methylbut-2-enyl) flavanone (77), sigmoidin B-4′-methyl ether (68) and 7,3′-dihydroxy-4′-methoxy-5′-(3-methylbut-2-enyl) flavanone (78) displayed respective IC_50_ values of 11.9, 11.4, 21.1, 12.9 and 10.7 μM (Trolox IC_50_ = 4.4 μM).^93^ The lower activity of 68 is likely related to its poor number of free hydroxyl groups.

### 6.3 Cytotoxic activity

The cytotoxic activity of a component is declared significant when the IC_50_ value is less than 10 μM or between 4–5 μg mL^−1^.^170^ Several reports claim that prenylated flavonoids have better anticancer effects than corresponding flavonoids and glycosides. It can be suggested that the hydrophilicity of these compounds could play an important role in the invasion of cancer cell lines.^171^ Several mechanisms of action are associated with the cell invasion, such as the initiation of apoptosis, the induction of autophagy, the inhibition of tumour angiogenesis and inhibition of cellular migration.^172^

Lysisteisoflavanone (113), erycaffra C (160), alpinumisoflavone (148), derrone (155), warangalone (141), isoerysenegalensein E (161), erysenegalensein E (151), laburnetin (165), senegalensein (23), isosenegalensein (162) and burttinone (28) exhibited a cytotoxic effect on human cervix carcinoma KB-3-1 cells, with respective IC_50_ values of 183, 104, 71.5, 230, 73.4, 99, 58.4, 250, 37.8, 53.8 and 58.8 μM, however, unfortunately no positive control was used during their assay.^130^ Erycaffra B (112) was reported to affect KB cells with an ED_50_ value of 12.3 μM.^63^ Lipoxygenase are expressed in tumour cells, epithelial and immune cells and play an important function in inflammation, skin disorders and tumorigenesis.^173^ Mildbone (33) and mildbenone (102) showed a moderate inhibitory effect on the enzyme with IC_50_ values of 41.8 and 59.7 μM relative to 22.6 μM of baicalein.^54^ Erymildbraedins A (166) and B (167), scandenone (141), erysenegalensein M (159), 5,4′-dihydroxy-2′-methoxy-8-(3,3-dimethylallyl)-2′′,2′′dimethylpyrano[5,6:6,7]isoflvone (168) and eryvarin B (173) were evaluated for their effect on the growth of MCF-7 breast cancer cells, LNCaP prostate cancer cells and Ishikawa endometrial cancer cells using MTT and/or SRB. Scandenone (141), 5,4′-dihydroxy-2′-methoxy-8-(3,3-dimethylallyl)-2′′,2′′dimethylpyrano[5,6:6,7]isoflvone (168) and eryvarin B (169) strongly inhibited the growth of MCF-7 breast cancer cells with EC_50_ values of 7.0, 6.8, and 7.1 μM, respectively. In addition, they inhibited the growth of Ishikawa endometrial cancer cells with EC_50_ values of 7.4, 7.4, and 7.7 μM, respectively. Compound 168 and 169 strongly affected the growth of LNCaP prostate cancer cells with EC_50_ values of 4.1 and 4.6 μM, respectively, while the activity of 141 was moderate (EC_50_ = 6.9 μM). In all cases, the EC_50_ values of the reference Faslodex® were between 7 and 30 μM.^53^ It was evident that the prenyl group at position 8 of these structures played a crucial role in their cytotoxic effect against these cells compared to other congeners where this prenyl was either oxygenated or the double bond was not at the same position. Phaseollidin (237) and cristacarpin (234) were assessed for their cytotoxicity towards several cancer cell lines and compound 237 exhibited an activity of 12.3 μM (no positive control).^85^ Erythribyssin A (260), erybraedin B (239), folitenol (264), erybreadin D (242), and erybreadin C (240) all showed cytotoxic effects on certain cancer cell lines (with the exception of erybreadin D (242) which had no effect on MCF7 and MDA-MB-231 or folitenol (264)); these compounds exhibited good to moderate cytotoxicity against MCF7 and MDA-MB-231 (human breast carcinoma cells), and the multidrug-resistant cell lines MCF7/TAMR and MCF7/ADR with IC_50_ values ranging from 5.6 to 28.0 μM, comparative to the positive control (Tamoxifen) which showed IC_50_ values in the range 10.9–12.4 μM.^145^ As an ongoing part of the same investigation, the authors evaluated the activity of erythraddisons I and II (208, 209), euchrenone b10 (174) and erysubin F (204) and erythraddisons III and IV (124, 125) and results showed that erythraddison II (209), erythraddisons III and IV (124, 125) and echrenone b10 (174) exhibited good cytotoxicity against MCF7 and MDA-MB-231 human breast carcinoma cells and the Adriamycin resistant cell line MCF7/ADR, with the IC_50_ values ranging from 4.32–11.41 μM (Tamoxifen, IC_50_ = 11.44, 11.13, and 12.41 μM, respectively).^78^ Neorautenol (244), phaseollin (236), calopocarpin (249), isoneorautenol (241), orientanol C (253) and cristacarpin (235) were studied for their effects on H4IIE rat hepatoma cells and phaseollin and neorautenol showed prominent toxicity on H4IIE cells, inducing apoptotic cell death at a dose of 2 μM.^79^ Sigmoidin A (6) was assessed for its cytotoxic effect on B16 melanoma and RAW 264.7 cell lines. It was found that this compound exhibited a dose-dependent cytotoxicity towards the two cells, with the higher activity observed at a concentration of 100 μM, which reduced the cell concentration to zero, compared to its congener eriodictyol with no prenyl fragments which inhibited less than 40% cytotoxicity on both cells. It appears from these results that the prenyl groups increase the cytotoxicity effect of flavonoids.^174^ Prenylated flavonoids isolated by Zarev *et al.* (2017) were evaluated for their antigenotoxic activities against aflatoxin B1 induced genotoxicity, and in the Vitotox assay, sigmoidin A (6), and B (7) showed good antigenotoxic activity with MIC values of 53.9 and 52.5 μM compared to curcumin (IC_50_ = 50 μM), however 4′-*O*-methylsigmoidin B (68), and abyssinins I (25), II (26), III (27) showed moderate activities with respective IC_50_ values of 68.1, 59.2, 68.1, and 61.4 μM.^84^ Sigmoidin I (118), sophorapterocarpan A (258), and 6α-hydroxyphaseollidin (259) induced apoptosis in Leukemia cells (CCRF-CEM) with IC_50_ values of 4.24, 3.73, and 3.36 μM, respectively in comparison to doxorubicin (IC_50_ = 0.20 μM). In addition, 6α-hydroxyphaseollidin (259) revealed good activity towards MDA-MB-231- pcDNA (breast cancer cells), HCT116 (p53+/+) (colon cancer cells), U87MG (glioblastoma cells) and HepG2 (Hepatocarinoma cells) with respective IC_50_ values of 5.70, 5.68, 4.71, and 6.44 relative to doxorubicin (IC_50_ = 1.1, 1.41, 1.06, 3.83 μM).^99^ Neobavaisoflavone (189), sigmoidin H (136), and isoneorautenol (241) were tested for their ability to affect the growth of certain cancer cell lines and isoneorautenol (241) exhibited a prominent cytotoxicity towards MDA-MB-231-BCRP (cDNA for the breast cancer resistance protein, BCRP) and knockout clones HCT116 (p53−/−) (colon cancer cells), with respective IC_50_ values of 2.67 and 9.89 μM compared to the positive control doxorubicin (IC_50_ = 7.83 and 4.06 μM).^108^ Alpinumisoflavone (148) and abyssinone V-4′-methyl-ether (18) showed a good binding affinity to ERα with an IC_50_ value of 4.5 μM, as well as to ERβ with IC_50_ = 15 μM for both compounds.^175^ Burttinone (28) exhibited a good cytotoxicity towards the colon cancer cell line HCC-2998 with an IC_50_ of 20 μM, however, no positive control was reported for the assay.^71^ Indicanines D (195), wighteone (197), alpinumisoflavone (148), erysenegalensein E (151) and 8-prenylerythrinin C (146) were assessed for their effects on human KB cells. They showed respective ED_50_ values of 12.5, 0.78, 4.13, 6.25, and 13.0 μg mL^−1^ (no positive control was reported).^69^ Excelsanone (215) and 6,8-diprenylgenistein (149) inhibited the DU145 prostate carcinoma cells at doses of 1, 10 and 20 μg mL^−1^, but only excelsanone (215) showed similar activity against PC3 prostate carcinoma cells.^9^ Addisoniaflavanones I (89) and II (90) reduced the viability of H4IIE hepatoma cells with respective EC_50_ values of 5.25 and 8.5 μM.^81^ The flavanones abyssinone V (5), 4′-methylabyssinone V (18), abyssinone IV (4), and abyssinoflavanone VII (43) showed good cytotoxicity with respective IC_50_ values of 15.0, 5.0, 15.0, and 3.5 μM. These values were good in comparison to those of their respective flavanone skeleton without prenyl groups (IC_50_ > 100 μM), illustrating the enhancement of the activity by the prenyl groups.^80^ Abyssinones A, C and D (106, 108 and 109) exhibited a cytotoxic activity against human colorectal cancer cell line Caco2, with IC_50_ values of 13.3, 15.1, and 11.1 μM, respectively. Abyssinone B (107) poorly affected these cells (IC_50_ > 30 μM).^128^ Erybraedin A (238), erythrabyssin II (235), phaseollin (236), eryzerin C (229), eriotrichin B (110), (6a*R*,11a*R*) 3-hydroxy-4(γ,γ-dimethylallyl)-2′,2′-(3′′-hydroxy)-dimethylpyrano[6′′,5′′:9,10]pterocarpan (287) and eryvarin D (255) showed cytotoxicity effects on human retinal endothelial cells (HRECs), with respective IC_50_ values of 4.21, 2.57, 3.65, 4.65, 5.85, 4.67 and 5.91 μM, compared to the positive control SH-11037 with IC_50_ = 0.018 μM.^142^

According to studies on molecular docking, the prenylflavonoids induced apoptosis by increasing the p53 protein. They are also believed to decrease the anti-apoptotic protein Bcl-2 and activate the caspase family in A549 cells. The prenyl groups attached to flavonoids interact with leucine, alanine, valine and lysine, which might be associated with the aforementioned apoptosis.^176^ Other studies have supported the up-regulation of the tumour necrosis factor-related apoptosis-inducing ligand and a down-regulation of the death receptor 5, thus contributing to the production of apoptotic amplificators.^177^

### 6.4 Anti-inflammatory activity

Prenylated flavonoids have been shown to have higher bioavailability than non-prenylated ones. The high pain-relieving effects of these flavonoids have therefore been associated with their bioavailability. Specific studies have been conducted on 8-prenylnaringenin and 8-prenylquercetin and these compounds were found to be accumulated in the liver, muscles and kidney.^178,179^ Other studies showed that icariin had a long term suppressive effect on paclitaxel-induced neuroinflammation and mechanical allodynia.^180^

Sigmodins A (6) and B (7) were tested against TPA-induced oedema and were all effective at a dose of 0.25 mg per ear by decreasing oedema by 89 and 83%, respectively, relative to the positive control indomethacin, which had a percentage of 83% at 0.5 mg per ear.^64^ The effect of erycristagallin (269) on ear inflammation induced by multiple topical applications of TPA was assessed and it inhibited swelling at 34% and the production of neutrophil infiltration at 59%, at a dose of 0.1 mg per ear.^64^ Abyssinone V-4′-methyl ether (18) was evaluated for its effects towards xylene induced-ear edema in mice and cotton pellet-induced granuloma model in rats; the best activity was obtained with a dose of 10 mg kg^−1^ of abyssinone V-4′-methyl ether (18), which inhibited the oedema at 71.43% compared to 2.5 mg kg^−1^ of dexamethasone (61.9% of inhibition) in xylene induced-ear edema in mice. This compound equally inhibited cotton pellet-induced granuloma model in rats at a dose of 10 mg kg^−1^ (61.32%) compared to dexamethasone at a dose of 2.5 mg kg^−1^ (68.72%).^181^ These pain-relieving activities are likely related to a high accumulation of the tested prenylated flavonoids in the muscles of the mice. The mechanism of action of these compounds might be related to the suppression of certain pro-inflammatory markers, including tumour necrosis factor α (TNF-α), interleukin 1β (IL-1β) and interleukin 6 (IL-6), and nuclear factor kappa-light-chain-enhancer of activated B cells (NF-κB) phosphorylation (p65) in the spinal cord of mice.^180^ Other findings support that this mechanism of action of prenylated flavonoids might be *via* the inhibition of cyclooxygenase-2 (COX-2), as demonstrated by the prenylated flavonoid cudaflavone B.^7^

### 6.5 Antiplasmodial activity

Abyssinone V (5), 5′-prenylpratensein A (93) and shinterocarpin (263) exhibited antiplasmodial activity with respective IC_50_ values of 4.9, 6.3, and 6.6 μM against the chloroquine-sensitive strain of *Plasmodium falciparum* (chloroquine IC_50_ = 0.008 μM). They equally displayed IC_50_ values of 6.1, 8.7, and 8.3 μM against a chloroquine-resistant strain (chloroquine IC_50_ = 0.075 μM).^75^ In addition, abyssinone IV (4) and erythrabyssin II (235) showed good antiplasmodial activities, with IC_50_ values of 7.7 and 6.5 μM against a chloroquine resistant strain (chloroquine IC_50_ = 0.093 μM) as well as 9.0 and 8.1 μM against a chloroquine sensitive strain (chloroquine IC_50_ = 0.008 μM).^88^ Abyssinin III (27), abyssinones IV (4) and V (5), sigmoidins A (6) and B (7) exhibited good antiplasmodial activity with IC_50_ values of 5.8, 5.4, 4.9, 5.8 and 8.1 μM against a chloroquine sensitive strain (chloroquine IC_50_ = 0.009 μM), as well as 5.2, 5.9, 6.1, 5.9 and 9.3 μM towards a chloroquine-resistant strain (chloroquine IC_50_ = 0.08 μM).^127^ Burttinol A (231) and C (233) and abyssinone V (5) displayed good activity with IC_50_ values 7.6, 9.3, and 5.7 μM against a chloroquine-sensitive strain (chloroquine IC_50_ = 0.009 μM) and 8.5, 9.1, and 6.6 μM against a chloroquine resistant strain (chloroquine IC_50_ = 0.08 μM), and Abyssinone V 4′-methyl ether (18) showed moderate activity (IC_50_ = 10.7 and 11.9 μM) against these respective strains, and in addition, the methyl at 4′ decreased its activity relative to abyssinone V (5).^182^

### 6.6 Antidiabetic activity

Overcoming insulin resistance is considered to be one of the major challenges for conventional anti-diabetic medication. The Protein Tyrosine Phosphatase 1B (PTP-1B) is recognized as a key element in the regulation of insulin signal transduction pathways, and is unfortunately considered to be a negative regulator of the insulin receptor pathway along with the leptin receptor pathway.^183^ Therefore, inhibiting this enzyme will contribute to overcoming insulin resistance.

The chemical constituents of *E. addisoniae* 5,2′,4′-trihydroxy-6-(γ,γ-dimethylallyl)-2′′′,2′′′-dimethyldihydropyrano[5′′′,6′′′] isoflavone (121), orientanol E (122), senegalensein (23), warangalone (141), warangalone 4′-methyl ether (205) and 2,3-dihydroauriculatin (117) were assayed for their inhibitory effect on the protein PTP1B, and orientanol E (122), 2,3-dihydroauriculatin (117) and 5,2′,4′-trihydroxy-6-(γ,γ-dimethylallyl)-2′′′,2′′′-dimethyldihydropyrano[5′′′,6′′′]isoflavone (121) exhibited the activities with IC_50_ values of 10.1, 2.6, and 4.1 μM, relative to the reference ursolic acid (IC_50_ = 2.5 μM). In contrast, the weak activity of other compounds might be related to the double 2,3.^77^ Compounds 45, 47, 49, 50, 53 and 54 exhibited good inhibitory effects on PTP1B with IC_50_ values of 13.9, 14.9, 18.2, 19.0, and 18.2 μM, respectively, compared to ursolic acid (3.6 μM).^90^ Additionally, compounds 51–2 and 40 were also assayed for their impact on the protein PTP1B. With the exception of 53 and 55 these metabolites showed dose-dependent activities, with IC_50_ values ranging from 15.2 to 19.6 μM compared to RK-682 (IC_50_ = 4.7 μM).^91^ Erylysin B (251), eryvarin D (255) and erybraedin A (238) also exhibited activity, with IC_50_ values of 6.0, 4.1, and 1.01 μM, respectively (ursolic acid IC_50_ = 2.5 μM).^70^ Neorautenol (244), erybreadin B (239), folitenol (264), erybreadin D (242), erysubin E (265) and erybreadin C (240) exhibited good activities against PTP1B protein with IC_50_ values of 7.6, 4.2, 7.8, 6.4, 8.8, and 7.3 μM, respectively (ursolic acid IC_50_ = 3.6 μM). Further investigation by Nguyen *et al.* in 2011 aimed to evaluate the activities of erythribyssin E (126), 5-deoxyabyssinin II (44), abyssinone III (3) 7-hydroxy-2-[4-methoxy-3-(3-methylbut-2-enyl)phenyl]chroman-4-one (72), abyssinone V (5), abyssinone II (2), prostratol C (128), erythribyssin G (73), erythribyssin I (74) and erythribyssin J (127) against PTP1B. Apart from erythribyssin I (74), the compounds exhibited moderate dose-dependent activities, with the IC_50_ range of 14.9–98.1 μM (IC_50_ = 3.6 μM for ursolic acid).^123^ Additionally, erythraddison II (211), euchrenone b10 (174) and erysubin F (203), as well as erythraddison III and IV (124, 125) showed good inhibitory effect on PTP3B, with the IC_50_ values ranging from 4.6–17.4 μM (ursolic acid: IC_50_ = 3.6 μM).^78^ Compound (3*R*)-2,7-dihydroxy-3-(3-methylbut-2-enyl)-2,2-dimethylpyrano[5,6:4,5]isoflavan (224) exhibited an IC_50_ value of 5.5 μM (ursolic acid: IC_50_ = 3.6 μM).^52^ Some compounds isolated from *E. mildbraedii* were evaluated for their inhibitory activity on the protein tyrosine phosphatase-1B (PTP1B) and abyssinone IV (4), and abyssinone VI-4′-*O*-methyl ether (101) potentially inhibited the activity of this protein with the respective IC_50_ values of 16 and 14.8 μM. Other compounds, abyssinone V-4′-*O*-methyl ether (18), abyssinone IV-4′-*O*-methyl ether (31), abyssinone V (5), sigmoidin E (10) and alpinumisoflavone (148) showed IC_50_ values of 26.3, 21.2, 39.7, 39.2, and 41.5 μM, respectively, with ursolic acid used as a positive control (IC_50_ = 3.6 μM). Regarding the activities of 4 and 101 compared to 18, 5, 10, and 148, the carbonyl in 4 and 101 could have improved the activity by chelating the hydrogen atoms in the protein.^51^ It is worthy to note that all the chemical structures of the compounds which exhibited antidiabetic activity beared the unmodified prenyl moieties.

### 6.7 Antiviral activity

8-Prenylluteone (139), auriculatin (152), erysenegalensein O (153), erysenegalensein D (150), erysenegalensein N (154), derrone (155), alpinumisoflavone (148) and 6,8-diprenylgenistein (149) were evaluated for their ability to inhibit the protease responsible for maturation of HIV-1 copies and they exhibited good to moderate activities with respective IC_50_ values of 4.0, 3.5, 5.0, 2.5, 4.5, 18.2, 30.1, and 0.5 μM compared to acetylpepstatin (IC_50_ = 0.09 μM). It was evidenced that the 6,8-diprenyl groups might improve the potency of HIV-1 PR inhibition in 4′-hydroxy isoflavonoids.^101^

### 6.8 Other activities

Erycaffrain A (150) showed moderate estrogenic activity with a MAC value of 1.25 μg mL^−1^ following the β-glucuronidase plant assay system.^163^ Clonic seizures were induced in mice by the ip injection of pentylenetetrazol (70 mg kg^−1^), picrotoxine (7.5 mg kg^−1^) and pilocarpine (375 mg kg^−1^); these animals then received injections of abyssinone V-4′-methyl ether (18). The best activities with this compound were recorded at 100 mg kg^−1^ in PTZ-induced (clonazepam, 0.1 mg Kg^−1^) and 25 mg kg^−1^ in PIC-induced (0.4 mg kg^−1^ clonazepam) and PILO-induced (Diazepam, 0.3 mg kg^−1^), thus illustrating the anticonvulsant effect of abyssinone V-4′methyl ether (18) *in vivo*.^96^ Erybraedin D (242) was evaluated for its ability to inhibit the enzyme 15-lipoxygenase, and displayed activity with an IC_50_ of less than 32 μM, while the positive control quercetin had an IC_50_ of 30 μM.^107^ 2,3-Dihydro-2′-hydroxyosajin (121), osajin (221) and 6,8-diprenylgenistein (143) showed hepatoprotective activities in CCl_4_ induced hepatotoxicity at respective percentages of 71.8, 67.54 and 69.41%, compared to 63.8% of silymarin.^106^

**Fig. 14:**
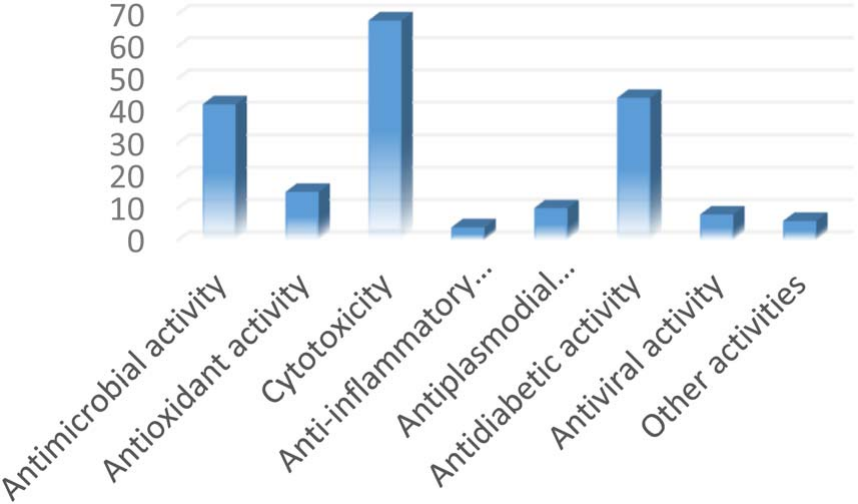
Summary of biological activities of prenylated flavonoids.

From studies on the biological activities of these prenylated flavonoids, it appears that a large number have exhibited cytotoxic effects towards cancer cell lines. The second most prominent activity was their antidiabetic and antimicrobial potential. However, few of these compounds were evaluated for their antiviral and anti-inflammatory activities. In addition, they were largely investigated for their antioxidant effect but had poor scavenging effects (Fig. 14). According to some specific subclasses, mainly isoflavones, pterocarpanes and flavanones exhibited cytotoxic activity. More than 20 flavanones derivatives contributed to antidiabetic effects of prenylated flavonoids occurring in the genus *Erythrina*. About 8 pterocarpans also showed antidiabetic potential. Overall, the prenylated flavonoids exhibited promising activities on certain bacteria, mainly *S. aureus* and *B. subtilis* (two Gram-positive bacteria), and 6,8-diprenylgenistein (149) exhibited a lower lethal dose (1 mg mL^−1^ or 2.46 mM) while showing good activity against *B. subtilis*, *E. coli* (Gram-negative bacteria) and *Candida mycoderma*.^72^ Unfortunately, not all these reported studies evaluated the toxicities of these antimicrobial compounds against normal cells. This is one of the challenges in terms of the search for lead or hit molecules, as many prenylated flavonoids were active. However, it is imperative that these studies be revised using state-of-the-art methodologies and analyses to re-evaluate these activities and carry out the cytotoxicity assays. Another challenge worth mentioning is the non-use of positive controls in certain assays, which renders the results less accurate. Certain methods used to assess biological activities in previous studies are obsolete and no longer in use. Hence, the results reported might not reflect the up-to-date challenges in drug discovery research.^184^ For example, the agar dilution method assay was not automated until recently. The well diffusion assay is well limited, and the 2,2-diphenyl-1-picrylhydrazyl (DPPH) free radical assay, ferric reducing antioxidant power (FRAP) assay and other methods of assessment of biological activity are no longer considered to be efficient.^184^ Various assays were only based on one method, whereas it is advised consider more than one *in vitro* assay to characterise the biological activity of a molecule^184^

This review therefore encourages more biological assays on prenylated flavonoids from *Erythrina* plants and other plant species. We also recommend multiple *in vivo* studies on the anticancer, antimicrobial, and antidiabetic activities of any prenylated flavonoids.

## 7 Some targeted proteins in drug analysis

### 7.1 Human epidermal growth factor receptor 2: 1N8Z

The HER2 is a protein belonging to the tyrosine kinase family that comprises more than one thousand amino acids. Its ligand-binding site contains 632 amino acids^185^ and does not bind any ligand as the other variants do (HER1, HER3, and HER4).^186^ This protein accounts for about 20% of breast cancers and 10 to 30% of gastric/gastroesophageal cancers.^187,188^ Jeong *et al.*, studying the breast cancer cell line SKBR3 reported that HER2 is co-localized with actin (phalloidin) in punctate regions of the plasma membrane that protrude from the apical aspect of these cells.^189^ The HER2 causes uncontrolled growth and division in breast cells, leading to breast cancer.^187,190^ Breast cancer can possess up to 25 to 50 copies of HER2 and up to 40 to 100-fold increase in HER2 protein, resulting in two million receptors expressed at the tumour cell surface.^191^ The activities of HER2 can be inhibited using tyrosine kinase inhibitors or monoclonal antibodies, thus suppressing the tumour cell growth.^192^ We should mention here that the variant HER1, generally referred to as EGFR1 or simply EGFR, is reported to be implicated in both lung and breast cancers,^193–195^ specifically, EGFR undergoes certain mutations to generate the subvariant EGFR TKI resistant;^194^ this protein has more affinity with ATP and its mechanism is thought to resist gefitinib or erlotinib^196^ and is encoded in PDB as 1M17.^197^

**Fig. 15:**
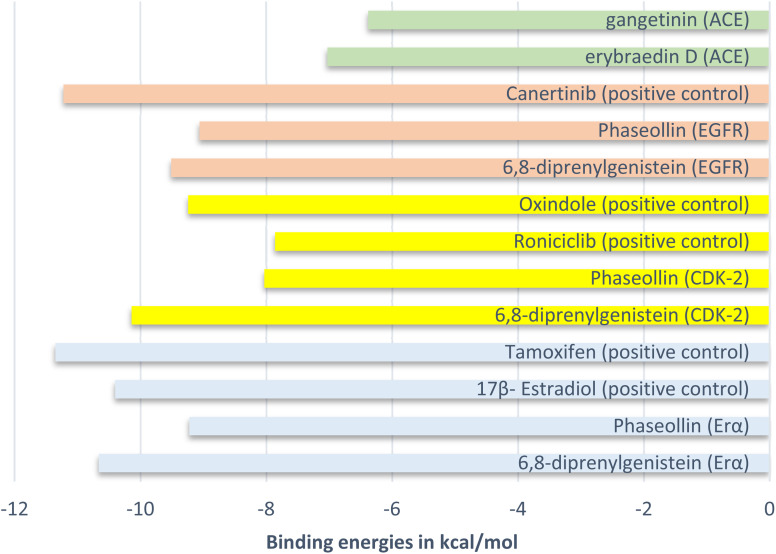
Binding energies of compounds 149, 236, 242 and 248.

6,8-Diprenylgenistein (149) and phaseollin (236) were docked *in silico* method for their ability to bind to the active site human oestrogen receptor- (hER-), B-cell lymphoma 2 (Bcl-2), cyclin-dependent kinase (CDK-2), ikappaB kinase (IkB) and growth factor receptor epidermal layer (EGFR); 6,8-diprenylgenistein (149) and phaseollin (236) showed binding energies with respective Δ*G* values of −10.66, and −9.22 kcal mol^−1^ (Fig. 15) with ERα receptor, compared to the positive controls 17β-estradiol (−10.40 kcal mol^−1^) and tamoxifen (−11.35 kcal mol^−1^); they equally showed respective binding affinities with CDK-2 receptor of −10.14, and −8.03 kcal mol^−1^ relative to roniciclib and 106 (oxindole) (Δ*G* = −7.86 and −9.24 kcal mol^−1^). These compounds also showed binding affinities with respective Δ*G* values of −9.51 and −9.06 kcal mol^−1^ with EGFR receptor in comparison to −11.22 kcal mol^−1^ for the positive control (canertinib).^198^

### 7.2 Angiotensin-converting enzyme 2: ACE2

Severe acute respiratory syndrome coronavirus 2 (SARS-CoV-2) is the causative agent of the pandemic COVID-19. The invasion of the host cells by the virus is facilitated by the Angiotensin-converting enzyme 2 (ACE2) receptor located at the surface of host cells.^199^ These receptors are expressed in various human organs.^200,201^ A recent study conducted by Herlina and coworkers revealed that erybraedin D (242) and gangetinin (248), out of 18 flavonoids from the genus *Erythrina*, had a better affinity with the ACE2 receptor.^202^ These two compounds were docked against the ACE2 receptor and had respective binding affinities of −7.028 and −6.379 kcal mol^−1^^202^ (Fig. 15). Therefore, these two flavonoids could play an important function in the pathogenicity of SARS-CoV-2.

## 8 Conclusion

In conclusion, twenty species of *Erythrina* were collected and investigated throughout Africa, and these studies resulted in the isolation and characterization of 289 prenylated flavonoids. A high percentage of these compounds proved to be prenylated flavanones. This is not surprising given that other subclasses of flavonoids derive from naringenin (flavanone subclass). According to the distribution, abyssinone V (5) and abyssinone V-4′-*O*-methyl ether (18), 6,8-diprenylgenistein (149) and phaseollidin (237) can be considered to be the chemical markers of the genus *Erythrina* as far as prenylated flavonoids are concerned. Pterocarpans were mainly characterized from *E. abyssinica*, *E. eriotricha*, *E. burtii* and *E. senegalensis*. No pterocarpan was isolated from *E. caffra* or *E. sigmoidea*. The flavanone subclass was largely characterized from *E. caffra*, *E. sigmoidea*, *E. latissimi*, *E. addisoniae* and *E. burtii*. Finally, the subclass isoflavone was mostly isolated from *E. caffra*, *E. abyssinica*, *E. burtii* and *E. senegalensis*. Most of these compounds have been evaluated for their biological effects, and were found to exhibit good antibacterial, anticancer and antidiabetic activities. About 68 prenylated flavonoids summarized in this work exhibited good cytotoxic effects against numerous cancer cell lines. 46 and 44 flavonoids exhibited promising antimicrobial and antidiabetic activities. Of these compounds, 20 prenylated flavanones displayed anti-diabetic properties. As some flavonoids are already marketed as dietary supplements for bacterial infections, antioxidant effect, immune system booster, prostatitis, and many more, the prenylated flavonoids herein reported have exhibited promising biological effects and therefore could be further investigated for their pharmacological activities.

The chemical shift ranges of different prenyl groups were highlighted to assist future structure elucidation or rapid identification of prenyl moieties. However, many prenyl groups possessing stereogenic centres were not fully characterized and further research will be needed to complete their structure elucidation.

## Author contributions

Conceptualization: B. Tsakem, X. Siwe-Noundou. Original draft preparation: B. Tsakem. Writing, review and editing: B. Tsakem, F. Ntie-Kang, R. B. Teponno, X. Siwe-Noundou. Supervision: F. Ntie-Kang, R. B. Teponno, X. Siwe-Noundou. Funding acquisition: F. Ntie-Kang, X. Siwe-Noundou. All authors have read and agreed to the published version of the manuscript.

## Conflicts of interest

The authors declare no conflict of interest.

## Supplementary Material

RA-015-D5RA03457D-s001

## Data Availability

The data supporting this article have been included in the manuscript and as part of the ESI.†
