# Supplementary material for: Naturally occurring prenylated flavonoids from African Erythrina plant species
Source: RSC Adv. 2025 Aug 18;15(34):27816–47. doi: 10.1039/d5ra03457d (PMC12377045; doi:10.1039/d5ra03457d)
Supplement: RA-015-D5RA03457D-s001 [file RA-015-D5RA03457D-s001.pdf]

Supplementary Information (SI) for RSC Advances.
This journal is © The Royal Society of Chemistry 2025

Naturally occurring prenylated flavonoids from African *Erythrina* plant species

Bienvenu Tsakem[a], Fidele Ntie Kang[b,c,d], Rémy Bertrand Teponno[e], Xavier Siwe Noundou[a,*]

Department of Pharmaceutical Sciences, School of Pharmacy, Sefako Makgatho Health Sciences University, Pretoria 0204, South Africa
Center for Drug Discovery, Faculty of Science, University of Buea, P.O. Box 63, Buea, Cameroon
Department of Chemistrym Faculty of Science, University of Buea, P. O. Box 63 Buea, Cameroon
Institute of Pharmacy, Martin-Luther University Halle-Wittenberg, Kurt-Mothes-Strasse 3, 06120 Halle (Saale), Germany
Department of Chemistry, Faculty of Science, University of Dschang, P.O. Box 67 Dschang, Cameroon

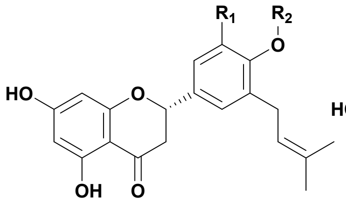

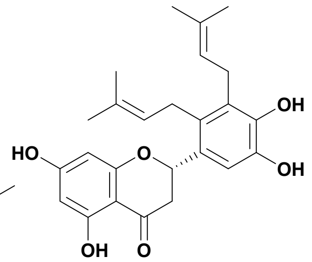

26 : R_1 = OCH_3, R_2 = H
34 : R_1 = H, R_2 = CH_3

27

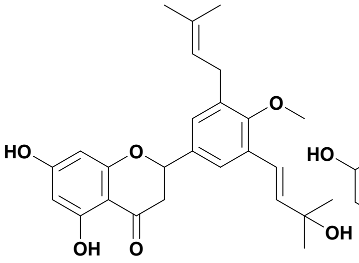

28

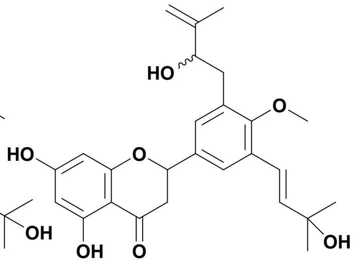

29

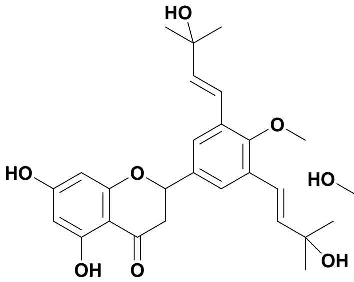

30

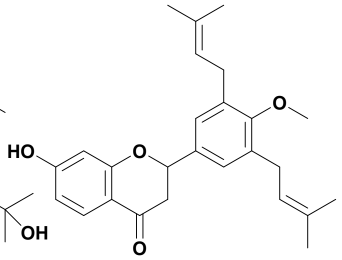

31

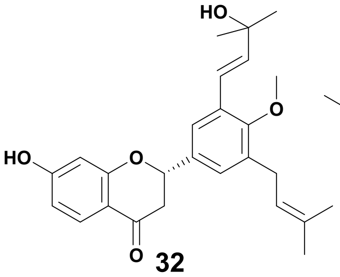

32

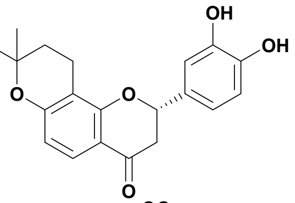

33

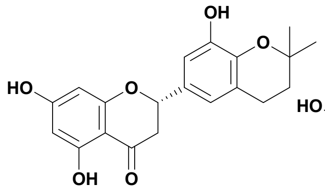

65 : R = H
66 : R = CH_3

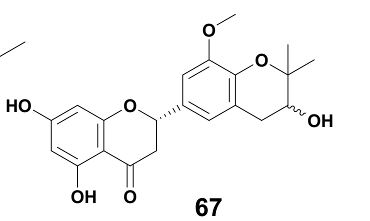

67

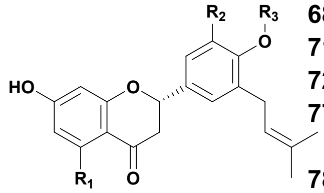

68 : R_1 = R_2 = OH, R_3 = CH_3
71 : R_1 = H R_2 = OH, R_3 = H
72 : R_1 = R_2 = H, R_3 = CH_3
77 : R_1 = OH R_2 = OCH_3, R_3 = CH_3
78 : R_1 = H R_2 = OH, R_3 = CH_3

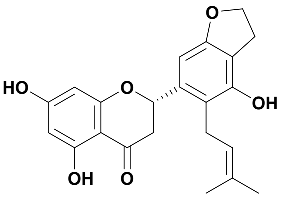

69

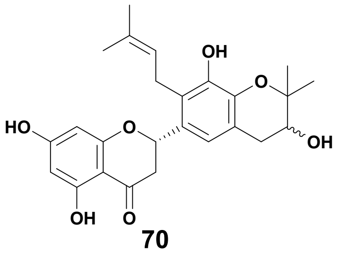

70

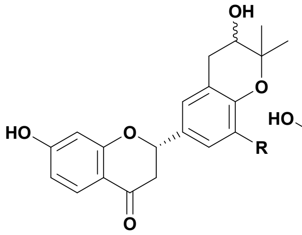

73 : R = Prenyl
74 : R = H

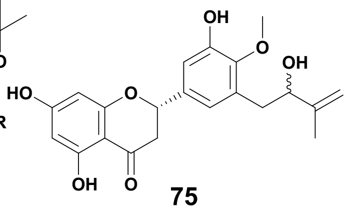

75

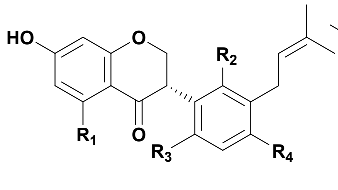

128 : R_1 = R_2 = H, R_3 = OH, R_4 = OCH_3
133 : R_1 = OH, R_2 = OCH_3, R_3 = H, R_4 = OH

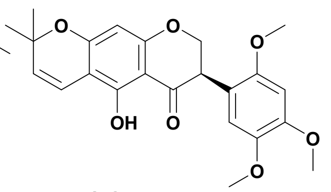

120

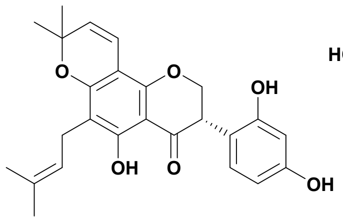

121

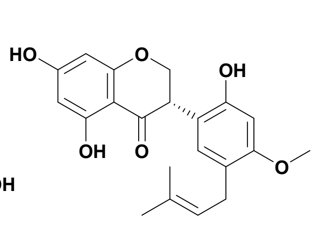

123

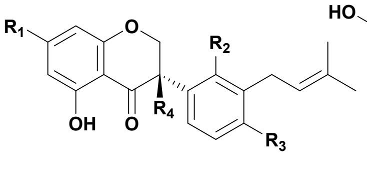

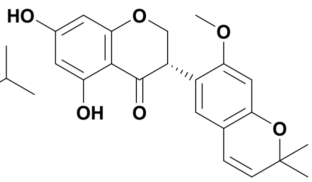

124 : $R_1 = R_2 = OH$, $R_3 = OCH_3$, $R_4 = H$

125

131 : $R_1 = R_3 = R_4 = OH$, $R_2 = OCH_3$

132 : $R_1 = R_2 = OCH_3$, $R_3 = R_4 = OH$

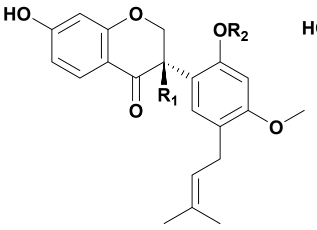

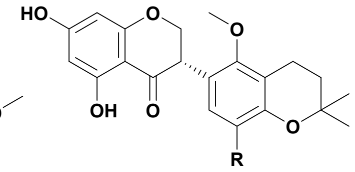

126 : $R_1 = H$, $R_2 = CH_3$

127 : $R_1 = OH$, $R_2 = H$

129 : $R = OCH_3$

130 : $R = H$

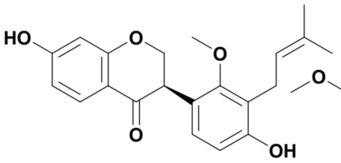

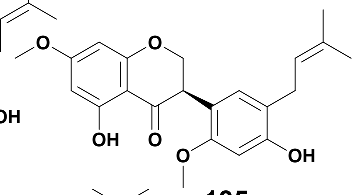

134

135

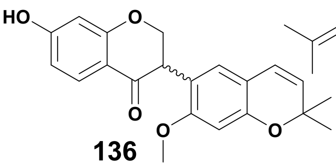

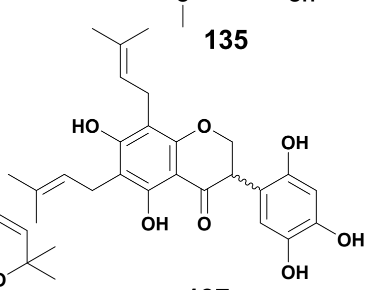

136

137

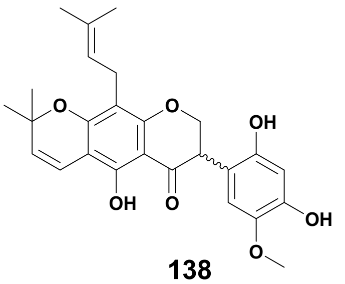

138

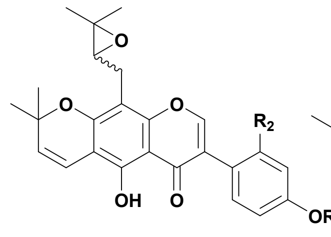

156 : R_1 = H R_2 = OH
157 : R_1 = H R_2 = H

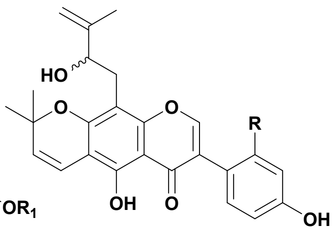

158 : R = OH
159 : R = H

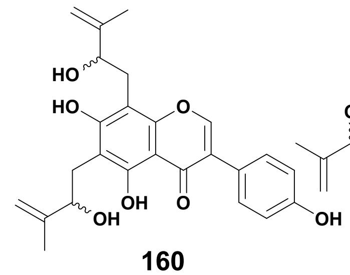

160

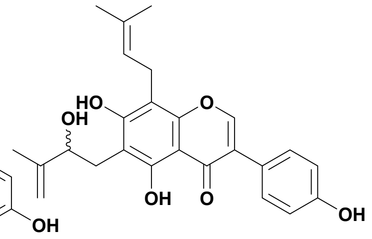

161

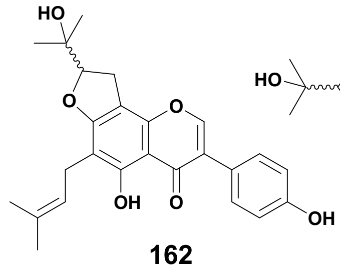

162

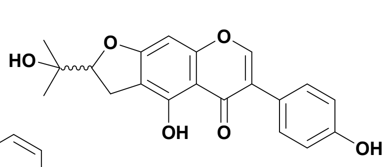

163

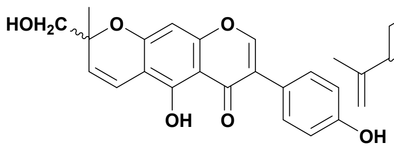

164

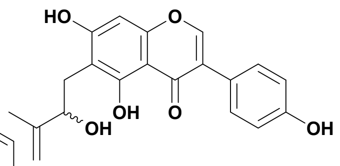

165

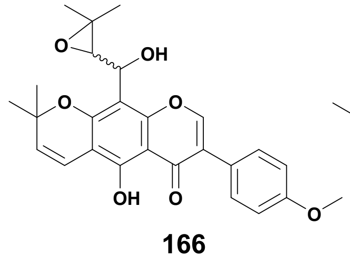

166

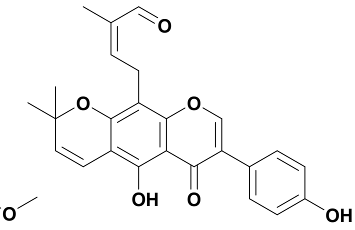

167

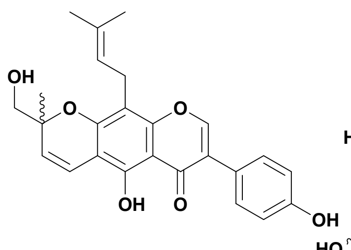

168

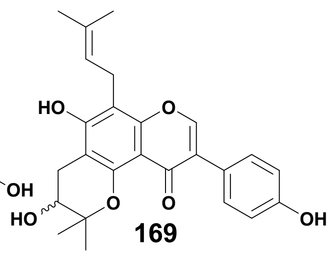

169

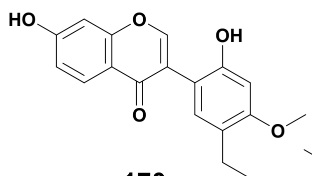

170

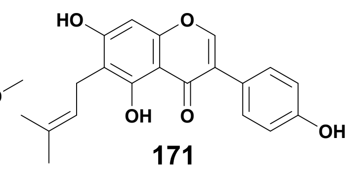

171

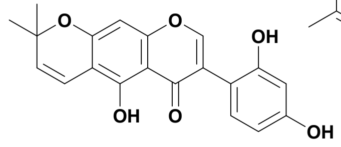

172

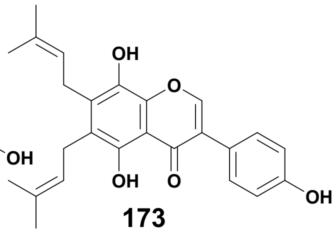

173

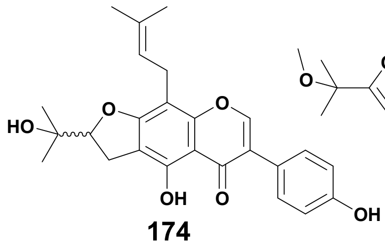

174

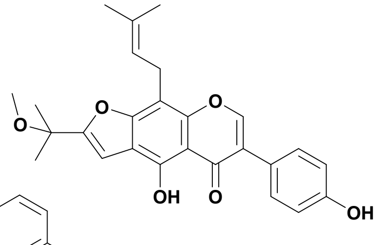

175

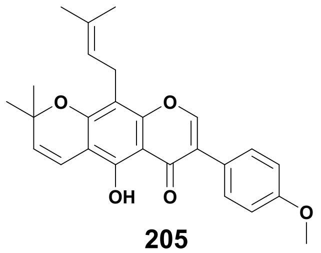

205

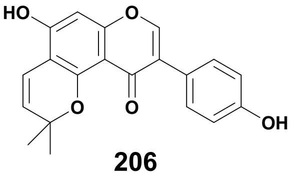

206

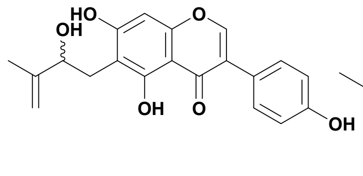

207

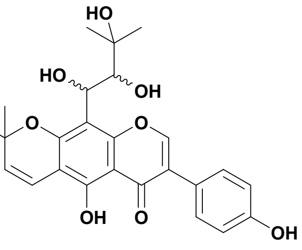

208

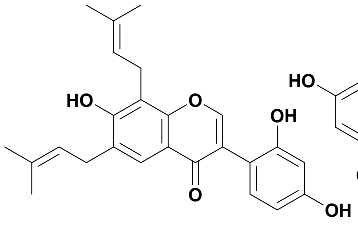

209

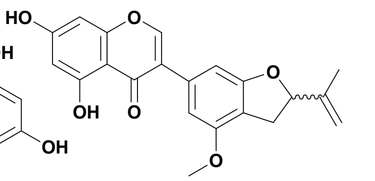

210

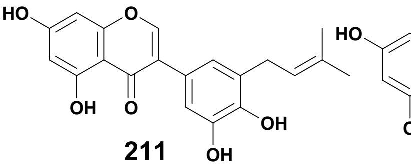

211

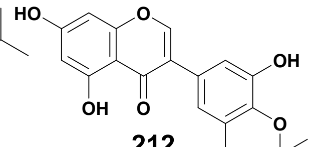

212

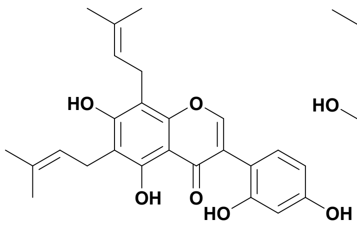

213

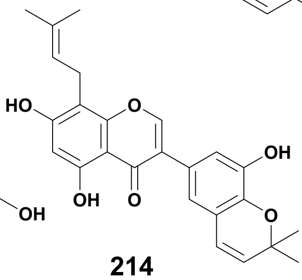

214

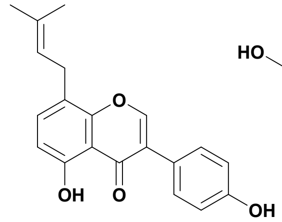

215

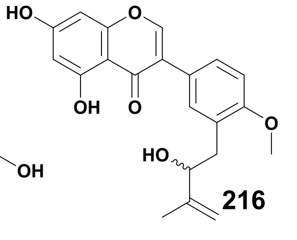

216

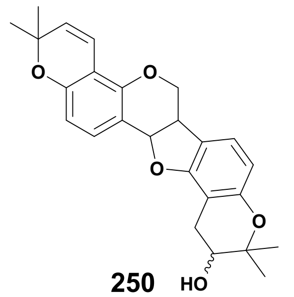

250

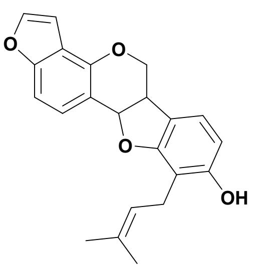

251

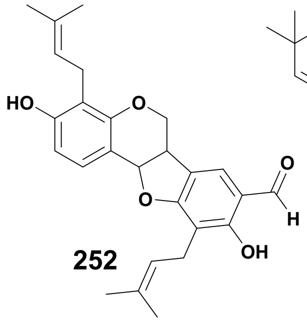

252

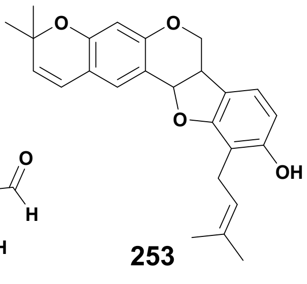

253

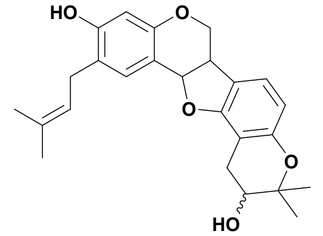

254

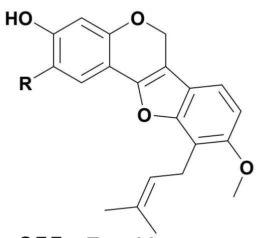

255 : R = H
289 : R = Prenyl

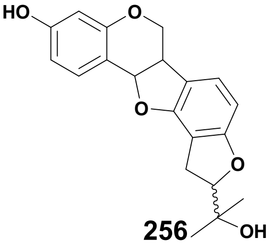

256

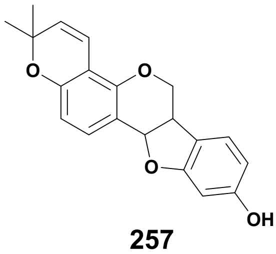

257

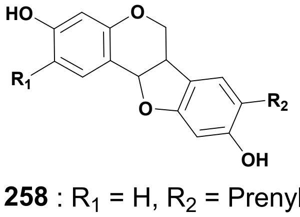

258 : R_1 = H, R_2 = Prenyl
263 : R_1 = Prenyl, R_2 = H

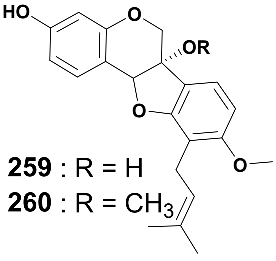

259 : R = H
260 : R = CH_3

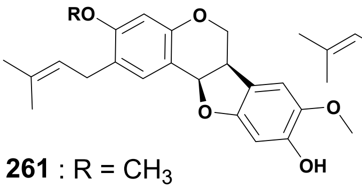

261 : R = CH_3
262 : R = H

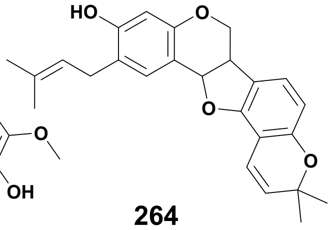

264

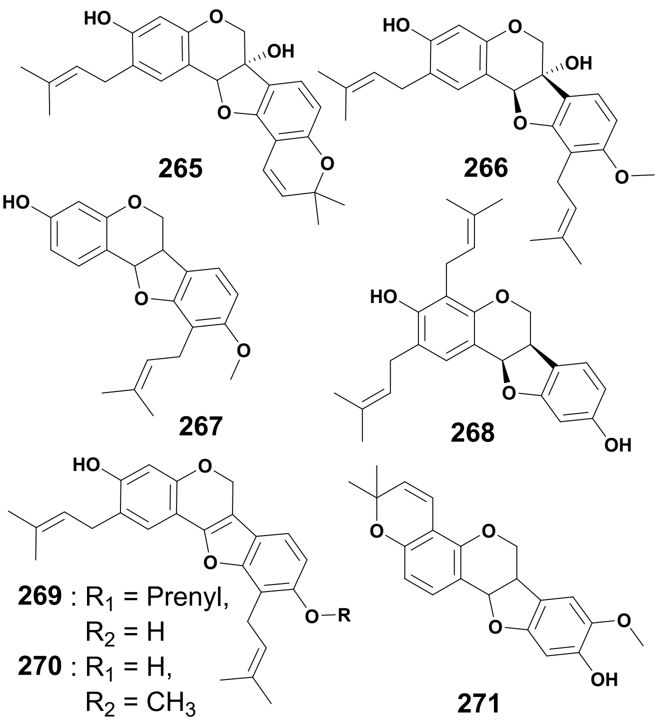

Fig. S1. Structures of prenylated flavonoids from the genus *Erythina* in Africa

Author contributions

Conceptualization: B. Tsakem, X. Siwe-Noundou. Original draft preparation: B. Tsakem. Writing, review and editing: B. Tsakem, F. Ntie-Kang, R. B. Teponno, X. Siwe-Noundou. Supervision: F. Ntie-Kang, R. B. Teponno, X. Siwe-Noundou. Funding acquisition: F. Ntie-Kang, X. Siwe-Noundou. All authors have read and agreed to the published version of the manuscript.

Conflicts of interest

The authors declare no conflict of interest.

Data availability

Not applicable.

Acknowledgements

The work reported herein was made possible through funding from the South African Medical Research Council through its Division of Research Capacity Development under the Research Capacity Development Initiative (RCDI) Programme to XSN. The content and findings reported/ illustrated are the sole deduction, view and responsibility of the researchers and do not reflect the official position and sentiments of the SAMRC XSN thanks the South Africa National Research Foundation / Competitive Support for Unrated Researchers (NRF/CSUR) Grant Number: SRUG2203291031. We acknowledge financial support from the Bill & Melinda Gates Foundation through the Calestous Juma Science Leadership Fellowship awarded to Fidele Ntie-Kang (grant award number: INV-036848 to University of Buea). FNK also acknowledges joint funding from the Bill & Melinda Gates Foundation and LifeArc (award number: INV-055897 and Grant ID: 10646) under the African Drug Discovery Accelerator program. FNK acknowledges further funding from the Alexander von Humboldt Foundation for a Research Group Linkage project.
